# Comparison of Amplitude‐ and Amplitude‐Frequency‐Modulated Pulses for Outer Volume Suppression in Cardiac MRI: Application to Myocardial $T_{2}$ Mapping

**DOI:** 10.1002/mrm.70515

**Published:** 2026-08-06

**Authors:** Ayda Arami, Maša Božić‐Iven, Christal van de Steeg‐Henzen, Yidong Zhao, Yi Zhang, Qian Tao, Alexandru Cernicanu, Hildebrandus Johannes Lamb, Sebastian Weingärtner

**Affiliations:** ^1^ Department of Imaging Physics Delft University of Technology Delft the Netherlands; ^2^ Department of Radiology Leiden University Medical Center Leiden the Netherlands; ^3^ Holland PTC Delft the Netherlands; ^4^ Philips Benelux Eindhoven the Netherlands

**Keywords:** amplitude‐frequency‐modulated (AM‐FM), outer volume suppression (OVS), reduced field of view (rFOV)

## Abstract

**Purpose:**

To evaluate single‐ and dual‐band pulse modules for suppressing extra‐cardiac signal in cardiac MRI, and test their applicability for time‐efficient imaging with reduced FOV in myocardial $T_{2}$ mapping at 3 T.

**Methods:**

Outer Volume Suppression (OVS) was implemented using two regional saturation slabs, along the phase‐encoding direction. Different Amplitude‐Modulated (AM) and Amplitude‐Frequency‐Modulated (AM‐FM) pulses were explored. AM sinc and AM‐FM Hyperbolic Secant (HS) pulses, as single‐ (S:1b and HS:1b) and dual‐band (S:2b and HS:2b) versions, were chosen for full evaluation. Pulses were individually optimized for minimal residual signal and optimal suppression‐band profiles. Pulse performance was assessed through simulations and phantom experiments, and evaluated in seven healthy subjects using residual signal and quantitative myocardial $T_{2}$ mapping with rFOV.

**Results:**

In simulations, optimized HS pulses demonstrated superior suppression‐band profile homogeneity and $B_{0}$ robustness, while both pulse classes showed comparable $B_{1}^{+}$ sensitivity. Phantom data revealed better signal suppression with HS pulses (S:1b: 19.22% ± 3.23%, S:2b: 16.85% ± 6.80%, HS:1b: 4.66% ± 1.04%, HS:2b: 2.85% ± 1.01%). The residual difference across slabs was slightly reduced using simultaneous dual‐band compared with sequential single‐band approaches (S:1b: 3.81% vs. S:2b: 0.92%; HS:1b: 4.56% vs. HS:2b: 0.66%). In vivo, despite significantly lower residual with HS pulses (*p* = 0.0015) and visually observed artifacts with sinc pulses, no significant difference in $T_{2}$ was observed across all configurations (*p* = 0.087) in myocardial $T_{2}$ mapping.

**Conclusion:**

AM‐FM pulses achieved robust suppression of extra‐cardiac signals, with dual‐band implementations minimizing suppression‐band differences. This can enable artifact‐free rFOV imaging, for improved image quality in myocardial $T_{2}$ mapping.

## 1 Introduction

Magnetic Resonance Imaging (MRI) of the heart offers the most comprehensive, one‐stop‐shop assessment of cardiac function and viability to date [1]. However, in cardiovascular MRI, the area of interest is considerably smaller than the required field of view (FOV) covering the entire chest. This necessitates trade‐offs between imaging resolution and acquisition duration.

Reduced FOV (rFOV) imaging has previously been proposed to ameliorate this trade‐off by suppressing signals within the FOV but outside the region of interest [2]. In Cartesian imaging, outer volume suppression (OVS) along the phase‐encoding (PE) direction can reduce the number of PE steps. This allows for shortening the scan time or increasing the resolution and slice coverage.

In clinical practice, OVS is most commonly implemented using image preparations with spatially selective saturation radio frequency (RF) pulses [3]. Typically, two 1D saturation pulses are applied sequentially to suppress both sides of the outer volume signal [3, 4, 5, 6, 7]. However, sequential application can lead to a variable amount of signal relaxation, especially for tissues with very short $T_{1}$ relaxation times, such as fat [7]. Dual‐band pulses can be used to suppress both sides along the PE direction simultaneously [8, 9], potentially homogenizing the suppression in the two bands. For example, cosine‐modulated dual‐band pulses have been investigated for simultaneous OVS of two slabs in the liver [10] and in fetal neuroimaging [3].

Effective OVS requires RF pulses with high selectivity, short duration, and high bandwidth. This is critical because saturation bands are placed near the edge of the FOV, where $B_{0}/B_{1}$ inhomogeneities are particularly pronounced. Conventional amplitude‐modulated (AM) pulses with linear‐phase exhibit incomplete saturation and insufficiently sharp band transitions at the large flip angles (FA) required for OVS [11]. Playing multiple OVS pulses of varying FAs, interleaved with gradient dephasing, improves saturation [3, 7]. However, this approach is limited to $90^{{}^{\circ}}$ FA, which is often suboptimal for cardiac imaging with linear readout due to signal recovery before k‐space center acquisition. The Shinnar‐Le Roux (SLR) design achieves selective profiles at arbitrary FA [5], but ties bandwidth to pulse duration, requiring longer pulses for $B_{0}$‐resilient performance [12]. Amplitude‐Frequency‐Modulated (AM‐FM) pulses, including hyperbolic secant (HS), have previously shown promise for OVS with improved resilience against field inhomogeneities [2, 13, 14]. In the sub‐adiabatic regime, these pulses enable arbitrary saturation FAs while delivering uniform slice profiles and decoupling bandwidth from pulse duration.

In this study, we sought to assess the effectiveness of different suppression pulses for use in OVS. Single‐ and dual‐band AM and AM‐FM pulses were evaluated and tested in rFOV imaging applied to myocardial $T_{2}$ mapping. Simulations were used to optimize pulse parameters, including suppression‐band FA and slice profile for all pulses. Next, phantom experiments were performed to assess the slice profile and the residual signal in the suppression‐band across all OVS pulses. Finally, in vivo experiments were carried out to quantify the residual suppression‐band signal and study the effectiveness of OVS in improving in‐plane resolution in the context of cardiac $T_{2}$ mapping.

## 2 Methods

Preparation‐based OVS was implemented with four classes of OVS pulses: AM and AM‐FM pulses, with either single‐ or dual‐band excitation. OVS was performed in two spatially selected bands along the PE direction in this study. The suppression‐band width was fixed at 150 mm on both sides. The total preparation duration, excluding spoiler gradients, was set to 20 ms to obtain comparable results across the pulses.

### 2.1 AM Pulses

**FIGURE 1:**
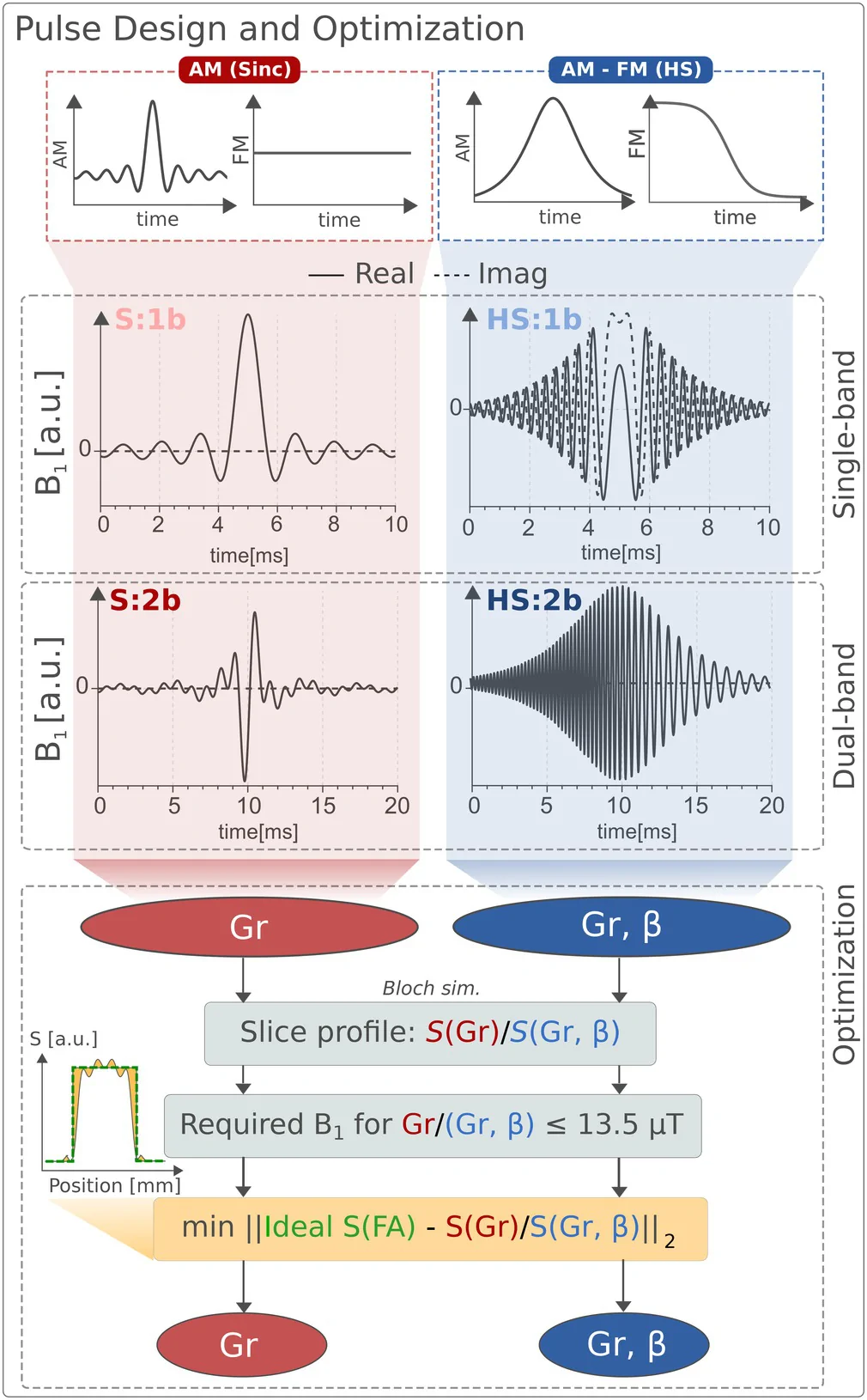
Pulse design and optimization. AM pulses (red) comprised of sinc pulses and AM‐FM (blue) of hyperbolic secant pulses with sech/tanh AM‐FM functions. Dual‐band pulses for both classes were shaped by frequency shifting the constituent single‐band pulses. For AM‐FM pulses, one of the bands was time‐reversed, resulting in a constant phase. Optimized parameters (Gr) for sinc and (Gr, β) for HS pulses were extracted separately for each of the four pulses using a homogeneous slice profile as the objective function. The maximum required $B_{1}^{+}$ was used as a boundary function, according to hardware limitations.

Sinc was chosen as the AM pulse for OVS in this study [15]. The time‐bandwidth product of the pulse was calculated from the fixed suppression‐band geometry and the pulse duration, fully characterizing the single‐band sinc (S:1b). Dual‐band sinc (S:2b) pulse was formed by combining two frequency‐shifted copies of the single‐band sinc (Figure 1). Additional AM pulses were generated using Shinnar‐Le Roux (SLR) pulse design [5] and compared with sinc pulses in terms of $B_{0}$/$B_{1}^{+}$ sensitivity, as described in the Supporting Information.

### 2.2 AM‐FM Pulses

HS pulses were used for OVS with AM‐FM modulation, as follows:

(1) $$A\left(t\right)=A_{0}\;\mathrm{sech}\left[\beta \left(1-\frac{2t}{\tau_{A}}\right)\right],\;0\leq t\leq \tau_{A},$$

(2) $$\omega \left(t\right)=\omega_{c}+\omega_{\max}\tanh\left[\beta \left(1-\frac{2t}{\tau_{A}}\right)\right],\;0\leq t\leq \tau_{A}.$$

Here, $\tau_{A}$ is the pulse duration, β determines the bell width, $\omega_{\max}$ defines the frequency sweep, and $\omega_{c}$ is the center frequency of the slab. As an AM‐FM alternative to HS pulse, SLR‐designed quadratic‐phase pulses were also investigated (see Supporting Information).

Dual‐band AM‐FM pulse (HS:2b) was constructed by combining frequency‐shifted single‐band AM‐FM (HS:1b). Here, one of the bands was time‐reversed prior to combination, leading to a constant pulse phase (Figure 1). HS dual‐band pulses were compared for the cases where the frequency modulation of the constituent pulses was swept in the same direction (concordant) or in the opposite direction by reversing one band (discordant). These experiments are described in the Supporting Information.

### 2.3 Optimization

#### 2.3.1 Suppression‐Band Flip Angle

The suppression‐band FA refers to the FA applied by the OVS pulse within the suppression‐band, while magnetization in the pass‐band ideally remains unaltered. The optimal suppression‐band FA was determined using Bloch simulations of the balanced steady‐state free precession (bSSFP) imaging sequence. The FA yielding the minimum residual signal for a predefined $T_{1}$/$T_{2}$ was chosen for optimal suppression of the extra‐cardiac signal. Simulations were performed with $T_{1}$ = 300 ms and $T_{2}$ = 50 ms to mimic subcutaneous fat at 3 T [16].

#### 2.3.2 Pulse Parameters

Optimization was performed by varying the relevant pulse parameters to obtain the best OVS performance for each pulse separately. For AM pulses, gradient strength (Gr) was optimized, while the effect of additional window functions for truncating the sinc pulses was investigated, as described in the Supporting Information. For AM‐FM pulses, both Gr and β were varied. The pulse parameters were optimized to achieve the smallest least‐squares deviation from the ideal, box‐shaped slice profile in single isochromat Bloch simulations. Optimization was performed under the boundary condition that the required $B_{1}^{+}$ amplitude does not exceed the hardware limit of 13.5 μT. The $B_{1}^{+}$ scaling required to achieve the desired suppression‐band FA was determined from the integral of the pulse shape for AM pulses and the analytical solution from configuration theory for HS:1b [17]. For HS:2b, it was obtained by numerically simulating the slice profile across a range of $B_{1}^{+}$ values.

Pulse performance in the presence of $B_{0}$/$B_{1}^{+}$ inhomogeneities was evaluated in simulations with optimized parameters. The $B_{0}$ offset was varied from −500 to 500 Hz in steps of 100 Hz, and the $B_{1}^{+}$ amplitude was varied between 5.5 and 13.5 μT with 0.5 μT step size.

### 2.4 Imaging Experiments

All imaging was performed on a 3 T Ingenia system (Philips, Best, The Netherlands). Phantom experiments used a torso‐shaped ballistic gel phantom ($T_{1}$ = 250 and $T_{2}$ = 40 ms, Fit Joe, Clear Ballistics, Greenville, SC, USA) for a realistic FOV.

To study pulse performance in phantom and in vivo, the residual signal was quantified in the suppression‐band for all optimized RF pulses. To this end, two ECG‐triggered snapshot bSSFP images were acquired: a baseline image without the OVS module and an image with the OVS module right before the imaging readout. A 6 s pause was played before the second image to ensure full longitudinal magnetization recovery. Other imaging parameters were as follows: Res. = 1.9 × 1.9 ${\mathrm{mm}}^{2}$, FA = 60°, TE/TR = 1.32/2.7 ms, Bandwidth = 1780.6 Hz/pixel, partial‐Fourier = 0.6, Number of PE Lines = 146, FOV = 300 × 282 ${\mathrm{mm}}^{2}$, slice thickness = 8 mm.

$T_{2}$ mapping was performed to study the use of OVS for rFOV imaging in a commonly used cardiac mapping sequence. A breath‐hold, ECG‐triggered, $T_{2}$‐prepared bSSFP sequence [18] was acquired with 3 dynamics: baseline (no preparation), $T_{2}$‐prepared image (TE = 45 ms, two HS, adiabatic refocusing pulses with duration of 10 ms and β = 1), and a saturation image using a 22.2 ms composite saturation pulse [19]. All image readouts were preceded by OVS modules. The remaining imaging parameters were set as listed for the bSSFP images above. For rFOV imaging, the FOV was reduced (300 × 141 ${\mathrm{mm}}^{2}$), and the imaging resolution increased (1.9 × 0.98 ${\mathrm{mm}}^{2}$), while keeping the shot duration fixed. $T_{2}$ maps were reconstructed in MATLAB using a 3‐parameter model [20].

#### 2.4.1 Healthy Subjects

The study was approved by the relevant local ethics committee (METC NL73381.078.20), and all participants provided written informed consent prior to the experiments.

The four OVS pulses were tested in seven healthy subjects (6M, 1F; 24.2±2.8 y/o). For each subject, the residual signal was quantified in suppression‐bands of the mid short‐axis (SAX) slice, following the same approach as in the phantom experiment. The suppression‐band was divided along the frequency encoding direction into three distinct ROIs: upper (ROI1), middle (ROI2), and lower (ROI3).

Variation in the FM function is known to alter the magnetization‐transfer effect (MT) [21], which may in turn influence the signal in the pass‐band. To investigate the MT contribution of each pulse, the signal change in the pass‐band was quantified with and without the OVS module for the blood pool and myocardium. For a given region of interest, the pass‐band modulation (PBM) was calculated as follows:

(3) $$\mathrm{PBM}=\frac{M_{0}-M_{\mathrm{OVS}}}{M_{0}},$$

where $M_{0}$ and $M_{\mathrm{OVS}}$ denote the signal acquired in the full field of view without and with the OVS pulse, respectively [22]. To minimize the contributions from residual side‐lobes, the ROI was chosen well outside the suppression bands. For reference, the PBM obtained in vivo was compared to the PBM obtained from measurements in a gel phantom with negligible bound‐pool to discern side‐lobe contributions from MT effects.

To evaluate OVS performance in subjects with a large amount of subcutaneous fat, peanut oil [23] was used to emulate extra‐cardiac signals. Two bottles of peanut oil were placed on the chest of healthy volunteers, and residual signal measurements were performed in two subjects (Two males; 26.5 ± 0.5 y/o).

Next, high‐resolution rFOV $T_{2}$ maps were acquired in three SAX slices (base, mid, and apex) with and without OVS. Finally, the full‐FOV, low‐resolution scan was obtained for reference. Template‐free group‐wise registration was applied to all dynamics within each slice [24, 25]. Automatic segmentation using a reverse imaging‐based cardiac MRI segmentation framework [26] with uncertainty estimation [27, 28] was applied. Mean and standard deviation of the $T_{2}$ values were extracted for the myocardium using the American Heart Association (AHA) 16‐segment model. The precision of myocardial $T_{2}$ measurements for each segment was assessed using the within‐subject coefficient of variation (wCV).

The difference in the average $T_{2}$ over three slices, with and without OVS, was evaluated with non‐parametric tests, due to the small sample size of the data. Group testing was performed using Friedman tests followed by pairwise Wilcoxon signed‐rank tests. The wCV for each segment and residual signal across all OVS pulses was statistically tested using the same group‐ and pair‐wise tests.

## 3 Results

### 3.1 Bloch Simulation

**FIGURE 2:**
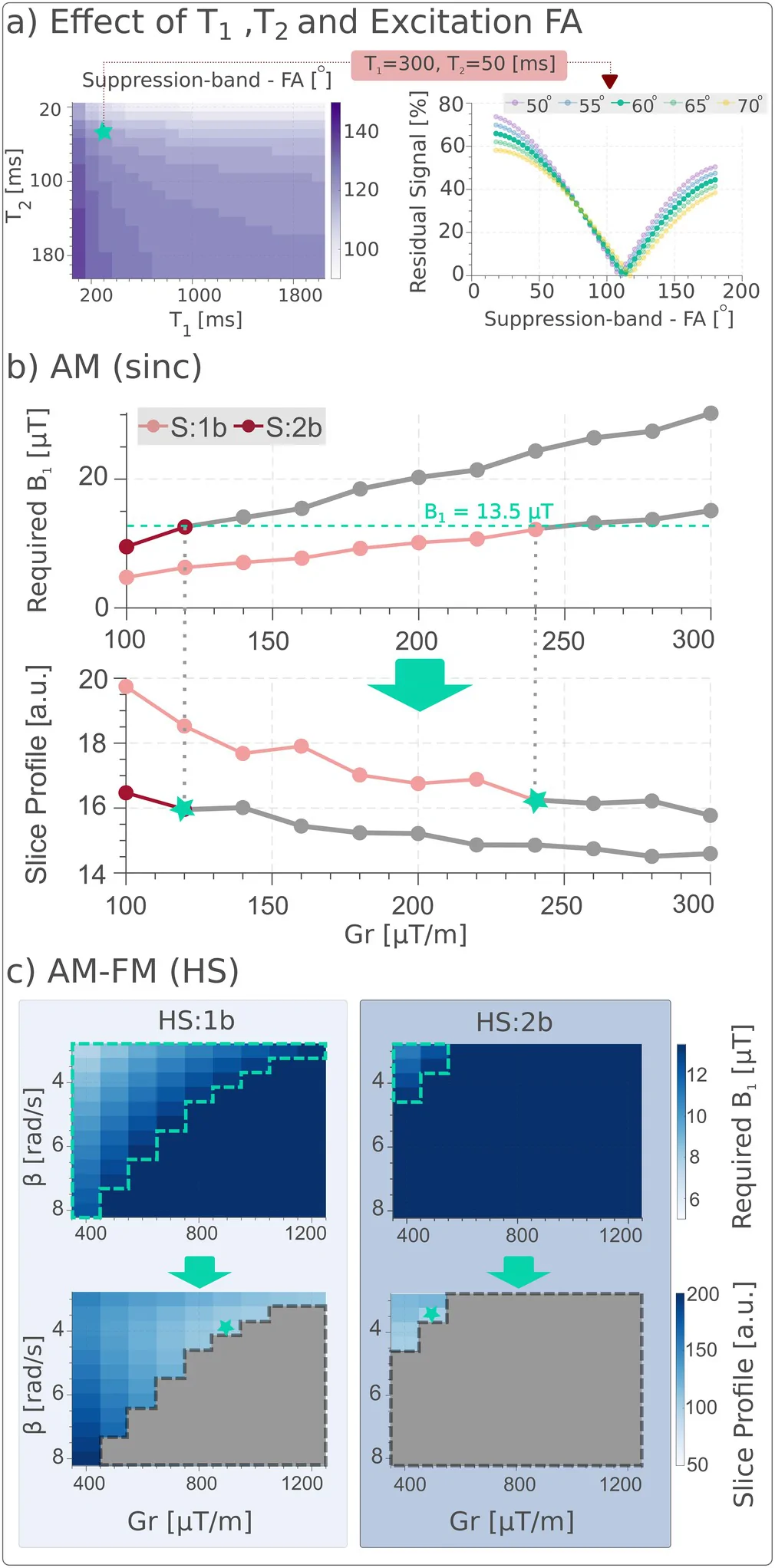
(a) Effect of $T_{1}$, $T_{2}$, and excitation flip‐angle (FA) on the optimal suppression‐band FA for optimal suppression with a bSSFP imaging readout with linear k‐space ordering. For subcutaneous fat ($T_{1}$ = 300 ms, $T_{2}$ = 50 ms, excitation FA = 60°), a suppression‐band FA of 120° yields minimal residual signal. The plot in the top right shows the residual signal as a function of the suppression‐band FA, for different excitation flip‐angles. The optimal suppression‐band FA is largely insensitive to excitation FA. (b) Pulse‐parameter optimization for sinc pulses. The top plot shows the $B_{1}^{+}$ required to realize the optimal suppression‐band FA as a function of the gradient strength (Gr). The bottom plot shows the deviation from an ideal slice profile for different Gr. Higher Gr achieves more homogeneous slice profiles, yielding Gr = 240 and 120μT/m as optimal pulse parameters within the $B_{1}^{+}$ constraint (≤13.5μT), for S:1b and S:2b, respectively. (c) The top plots show the required $B_{1}^{+}$ for various β and Gr combinations for HS pulses. The bottom plots show the agreement with the ideal slice profiles for allowed conditions. In this case, $B_{1}^{+}$ constraints limited parameters to (β = 3.5, Gr = 1000 μT/m) for HS:1b and (β = 3.5, Gr = 500 μT/m) for HS:2b.

In simulations, optimal suppression was achieved for subcutaneous fat at 3 T ($T_{1}=300$ and $T_{2}=50$ ms) with a suppression‐band FA of approximately $120^{{}^{\circ}}$ (Figure 2a). Window functions affected the slice profile but had a negligible effect on the OVS‐relevant performance criteria of $B_{0}$ and $B_{1}^{+}$ resilience, as shown in Figure S2. The optimal suppression‐band FA was largely insensitive to the excitation FA, ranging between 113.9° at 50° and 123.8° at 70°. The most homogeneous slice profiles within hardware limits ($B_{1}^{+}\leq 13.5$ μT) were obtained for Gr of 240 and 120 μT/m for S:1b and S:2b, respectively (Figure 2b). For AM‐FM pulses β=3.5 with Gr of 1000 μT/m yielded the best results for HS:1b, and β=3.5 and Gr of 500 μT/m for HS:2b (Figure 2c).

**FIGURE 3:**
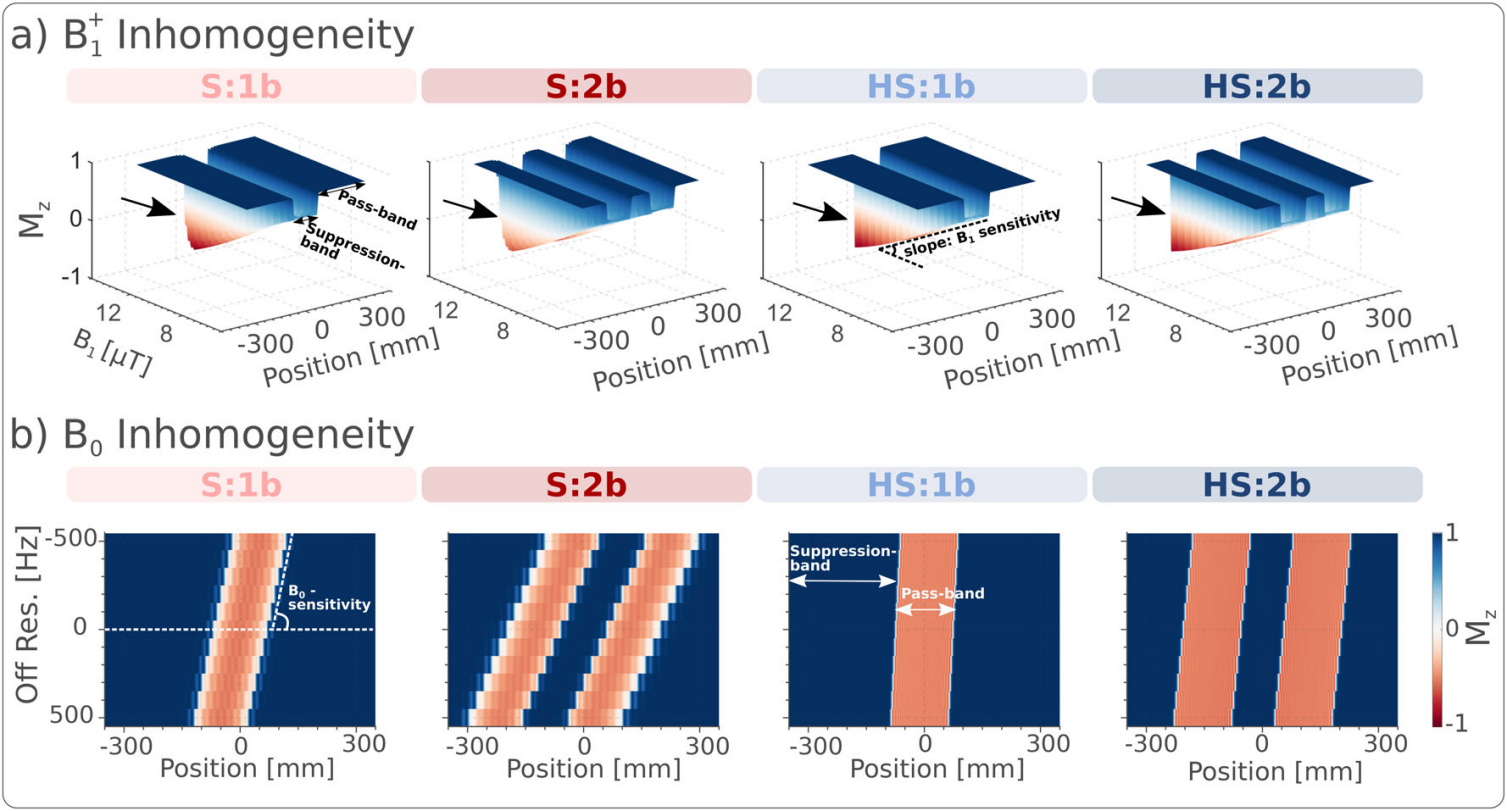
(a) Bloch‐simulation‐derived suppression‐band profiles of the four different pulse types for varying $B_{1}^{+}$ scaling. Sinc and HS pulses exhibited comparable sensitivity to $B_{1}^{+}$ inhomogeneity, evidenced by a similar slope. HS pulses produced sharper pass‐band‐to‐suppression‐band transitions (black arrows) compared with sinc pulses. (b) Suppression‐band profile of the pulses in the presence of off‐resonance. HS pulses demonstrated greater resilience to $B_{0}$ inhomogeneity (larger slope) as well as more homogeneous slice profiles.

Figure 3a,b presents the simulated performance of all pulses with optimized parameters for different $B_{1}^{+}/B_{0}$ inhomogeneities. Overall, HS pulses achieved more homogeneous slice profiles with noticeably sharper pass‐band‐to‐suppression‐band transitions compared with sinc pulses. HS and sinc demonstrated comparable $B_{1}^{+}$ sensitivity (Figure 3a). Both pulse types exhibited similar changes in suppression‐band FA as $B_{1}^{+}$ varied, with only marginally better performance in HS pulses: S:1b ($R^{2}$ = 0.99, slope = −0.16, intercept = 1.47), S:2b ($R^{2}$ = 0.99, slope = −0.15, intercept = 1.44), HS:1b ($R^{2}$ = 0.98, slope = −0.14, intercept = 1.33), HS:2b ($R^{2}$ = 0.98, slope = −0.13, intercept = 1.33). In the presence of $B_{0}$ inhomogeneity, HS pulses achieved improved robustness with reduced band shifts. Among HS pulses, HS:1b showed slightly better $B_{0}$ robustness. (Figure 3b).

Additional comparisons, including SLR‐designed pulses, showed that SLR‐designed AM pulses were nearly indistinguishable from sinc pulses in sensitivity to $B_{1}^{+}$ scaling (${\mathrm{dM}}_{z}$/$B_{1}^{+}$ between −0.14 and −0.15 vs. −0.14 for sinc) and comparable in maximum achievable bandwidth following the optimization (1400 vs. 1532 Hz) (see Figure S3). Single‐band SLR‐designed AM‐FM pulses with quadratic‐phase achieved higher bandwidth in optimization than HS:1b while maintaining comparable $B_{1}^{+}$ sensitivity. The dual‐band quadratic‐phase pulses, however, showed substantial pass‐band ripples despite having comparable bandwidth to HS:2b (see Figure S4).

### 3.2 Phantom and In Vivo Imaging

**FIGURE 4:**
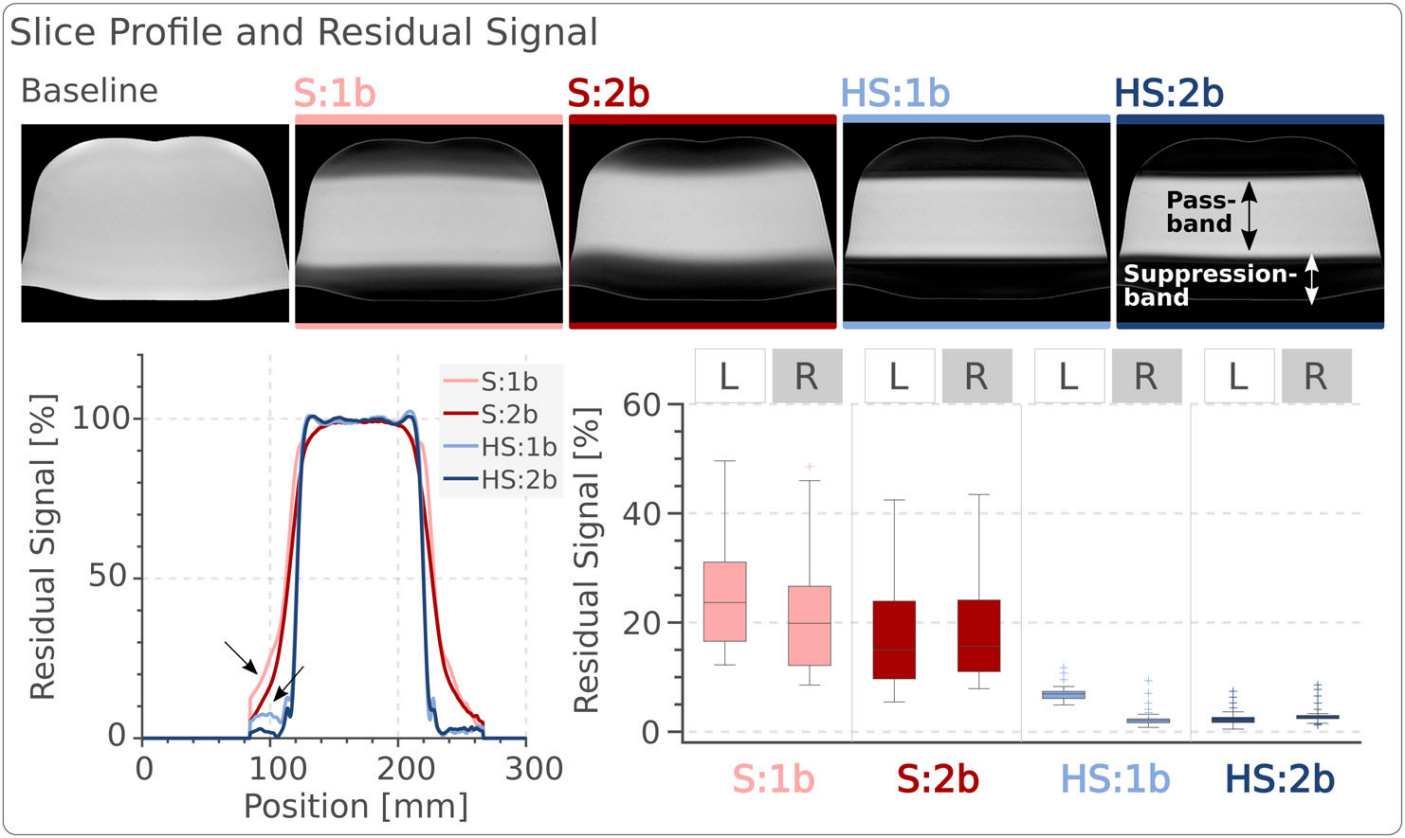
Suppression‐bands and residual signals in both bands in phantom measurements. Dual‐band pulses achieved more homogeneous suppression across bands, yielding a smaller residual signal difference between the two bands compared with their single‐band counterparts (black arrows).

The slice profile measurements obtained in the phantom showed good agreement with Bloch simulation results (Figure 4). Residual signal in the phantom demonstrated superior performance for AM‐FM pulses compared with AM approaches. The different pulses yielded the following residuals: S:1b: 19.22% ± 3.23% and S:2b: 16.85% ± 6.80%, HS:1b: 4.66% ± 1.04% and HS:2b: 2.85% ± 1.01%. Single‐band and dual‐band pulses showed differences in the homogeneity between the two suppression‐bands. The sequential excitation for single‐band pulses led to differences in the residual signal of two slabs of 3.81% for S:1b and 4.56% for HS:1b compared with 0.92% for S:2b and 0.66% for HS:2b.

**FIGURE 5:**
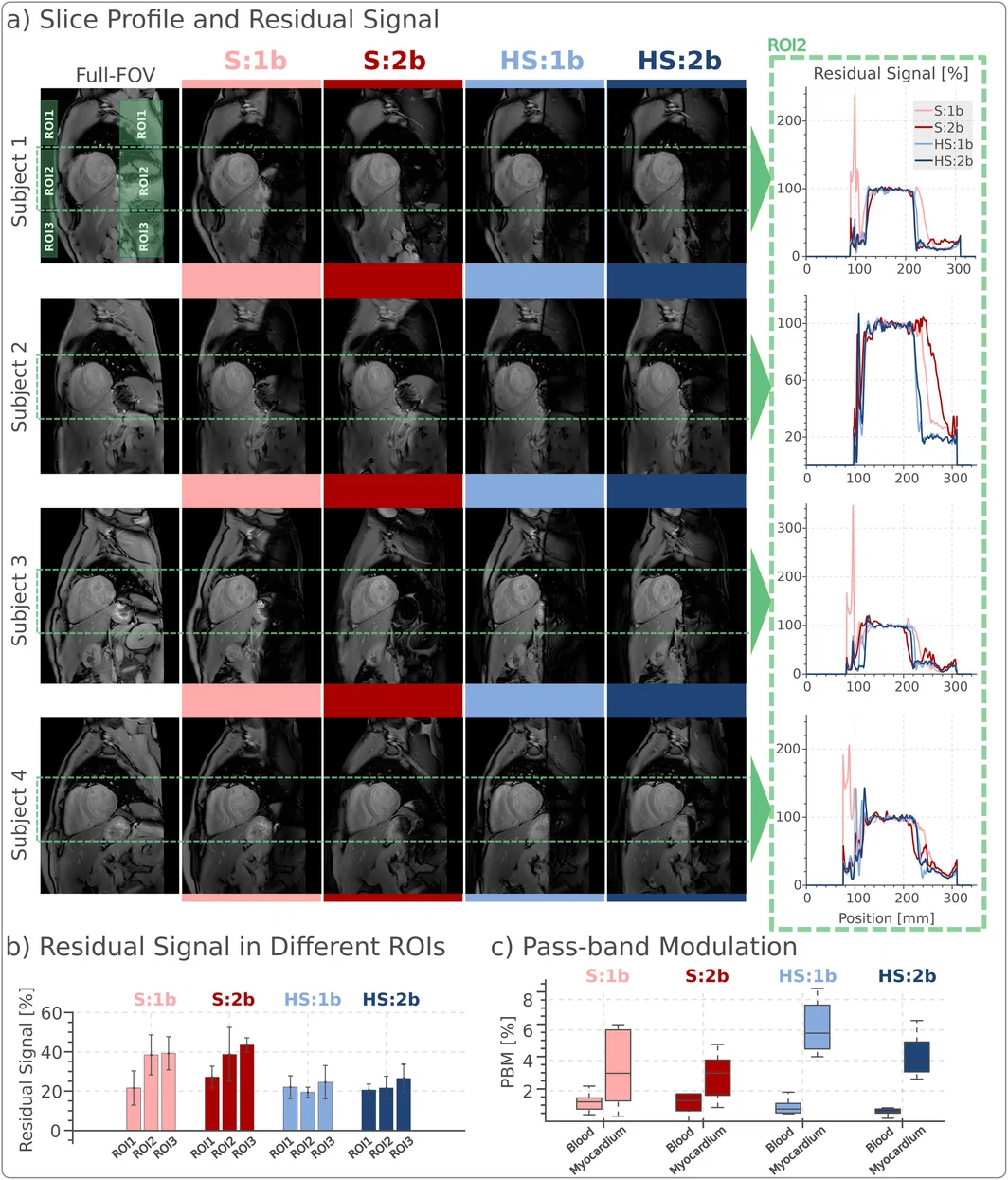
(a) In vivo images of suppression bands for all OVS pulses in four representative subjects. Sinc pulses (S:1b and S:2b) exhibited larger distortion and angulation of the pass‐ to suppression‐band transition, which remained well delineated with HS pulses (HS:1b and HS:2b). (b) Residual signal in 3 ROIs across all volunteers for all pulses shows lower suppression performance for sinc pulses. (c) Pass‐band signal modulation in blood and myocardial ROIs for all four pulse classes. Negligible modulation is observed in the blood pool, as expected from the minimal MT effect, and moderate modulation in the myocardium.

In vivo images displaying the suppression‐bands obtained with different pulse modules show overall largely successful cancellation of the extra‐cardiac signal, particularly in the posterior region (Figure 5a). However, AM pulses show distorted and angulated pass‐band‐to‐suppression‐band transition. The corresponding suppression‐band profiles show a trend of overall higher residual signal than observed in the phantom. Across all subjects, AM‐FM pulses show consistently improved suppression compared with AM approaches (ROI2: group‐wise *p* = 0.015, and $p_{\text{pairwise}}\leq 0.031$; S:1b: 28.80% ± 7.94% and S:2b: 37.11% ± 31.38%, and HS:1b: 17.32% ± 3.86% and HS:2b: 19.03% ± 5.90%). Across the three ROIs (Figure 5b), ROI3 yielded the highest residual signal for all subjects and pulse types (S:1b: 39.23% ± 8.44%, S:2b: 43.51% ± 3.62%, HS:1b: 24.50% ± 8.50%, HS:2b: 26.4% ± 7.20%). However, no significant differences were observed between the HS:1b and HS:2b (*p* = 0.062) or S:1b and S:2b pulses (*p* = 0.312). Phantom and in vivo results showed that sweep direction had a negligible effect on HS:2b pulse performance in this study (Figures S7 and S8). Furthermore, no significant differences were found between SLR‐designed quadratic‐phase pulses and HS pulses (Figures S5 and S6), except for the dual‐band SLR pulse, which showed noticeable ripples in the pass‐band.

Figure 5c presents the PBM across all pulse types in the myocardium and blood for all volunteers. In the blood pool, negligible PBM was measured across all pulse types (S1:1b [1.09% ± 0.69%], S:2b [0.87% ± 0.68%], HS:1b [0.83% ± 0.46%], HS:2b [1.06% ± 0.76%]). The PBM in the blood pool is closely in line with the PBM obtained in a largely MT‐free phantom (S:1b 1.02%, S:2b 0.97%, HS:1b 0.54%, HS:27b 0.2%), indicating minor MT effects in both settings. In the myocardium, moderate PBM was observed for AM pulses (S:1b: [3.23% ± 1.92%], S:2b [2.41% ± 1.64%]). AM‐FM pulses showed higher PBM values, particularly when performed as a single‐band pulse (HS:1b [5.03% ± 2.12%], HS:2b [3.81% ± 1.35%]).

**FIGURE 6:**
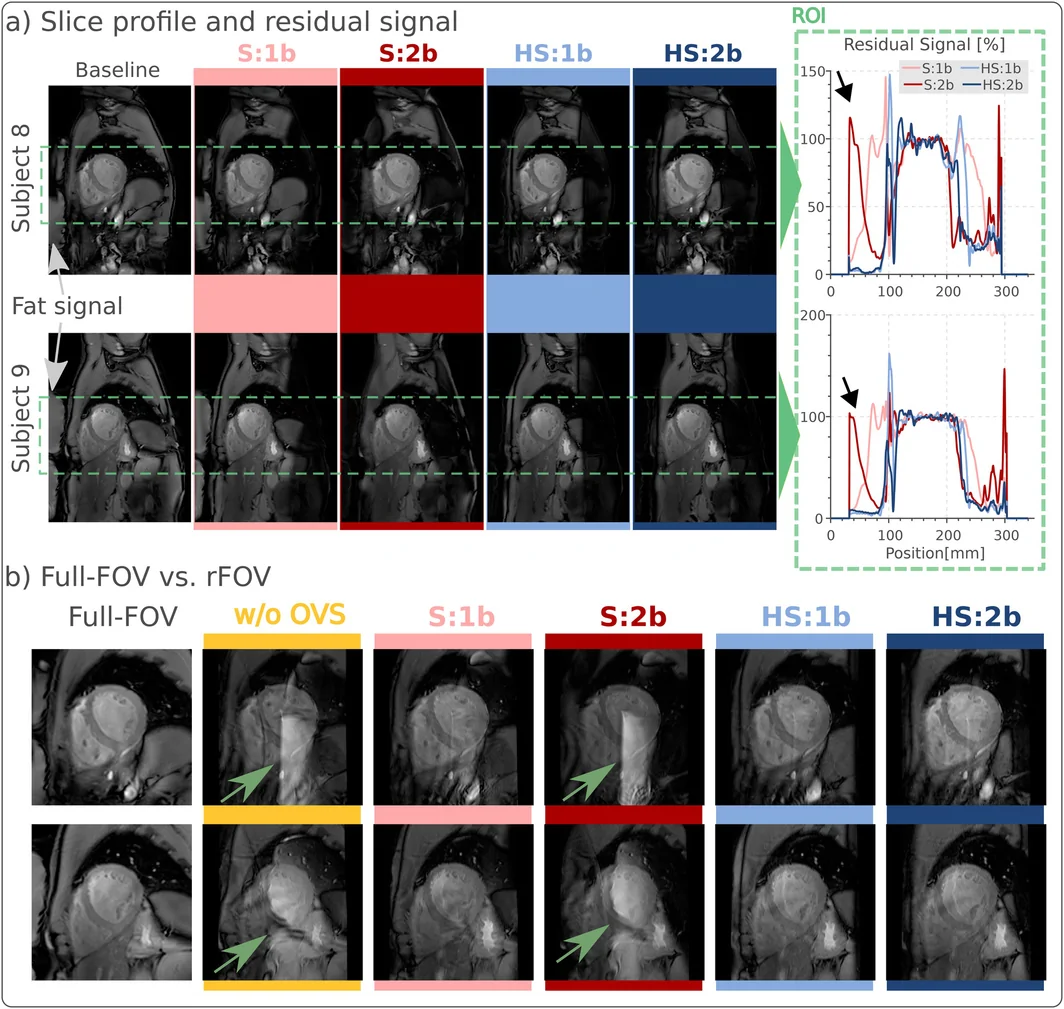
(a) Suppression‐band and residual signal for all OVS pulses in two subjects, with two peanut oil bottles on the chest to mimic thick subcutaneous fat (gray arrows). AM pulses (S:1b, S:2b) show substantial residual signal in the anterior region, which is largely suppressed for AM‐FM pulses (HS:1b, HS:2b). (b) Full‐FOV and rFOV images with and without OVS. Visually disruptive fold‐over artifacts (green arrows) can be observed without OVS and AM pulses, especially S:2b, but are markedly alleviated with HS.

Figure 6a illustrates the suppression‐bands and residual signal acquired in subjects where peanut oil bottles were placed on the chest of the subject to emulate subcutaneous fat. Dominant residual signal is observed in the anterior region for the AM pulses, which was largely alleviated with AM‐FM pulses (S:1b 48.5%±9.89%, S:2b 38.46%±4.61%, HS:1b 10.57%±3.39%, HS:2b 11.21%±2.41%). Reduced FOV images acquired in the subject with emulated subcutaneous fat show visually disruptive image artifacts if no OVS is applied (Figure 6b). AM pulses fail to sufficiently suppress the dominant fold‐over artifact, even if applied as a single‐band pulse, whereas AM‐FM pulses achieve substantially lower residual signal.

**FIGURE 7:**
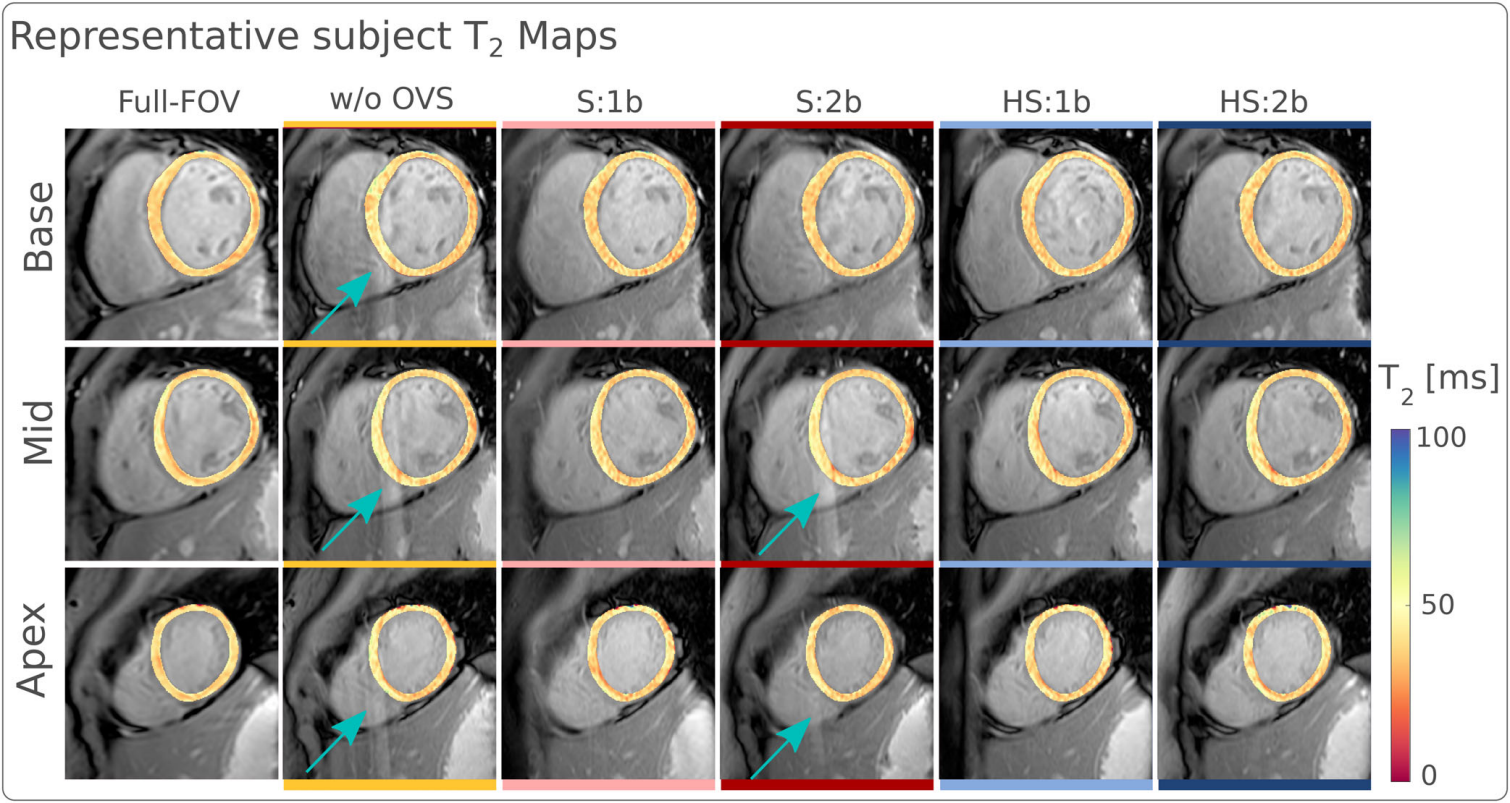
$T_{2}$ maps obtained with rFOV for all pulses and without OVS, in comparison with full‐FOV as a reference. $T_{2}$ maps show superior signal suppression with no visually apparent artifacts (teal arrow) when using OVS with HS pulses.

Figure 7 depicts myocardial $T_{2}$ maps in 3 SAX slices for full‐FOV, rFOV with and without OVS in the representative subject. Substantial aliasing artifacts were observed in the septal area of the mid and apical slices when no OVS was used (Figure 7). Aliasing artifacts were significantly reduced using OVS pulses, with only S:2b showing visually discernible residual artifacts. The artifact reduction was most pronounced for AM‐FM pulses. The reconstructed myocardial $T_{2}$ values showed visually improved homogeneity and agreement with the reference scan (full‐FOV) when OVS was used compared with rFOV without OVS.

**FIGURE 8:**
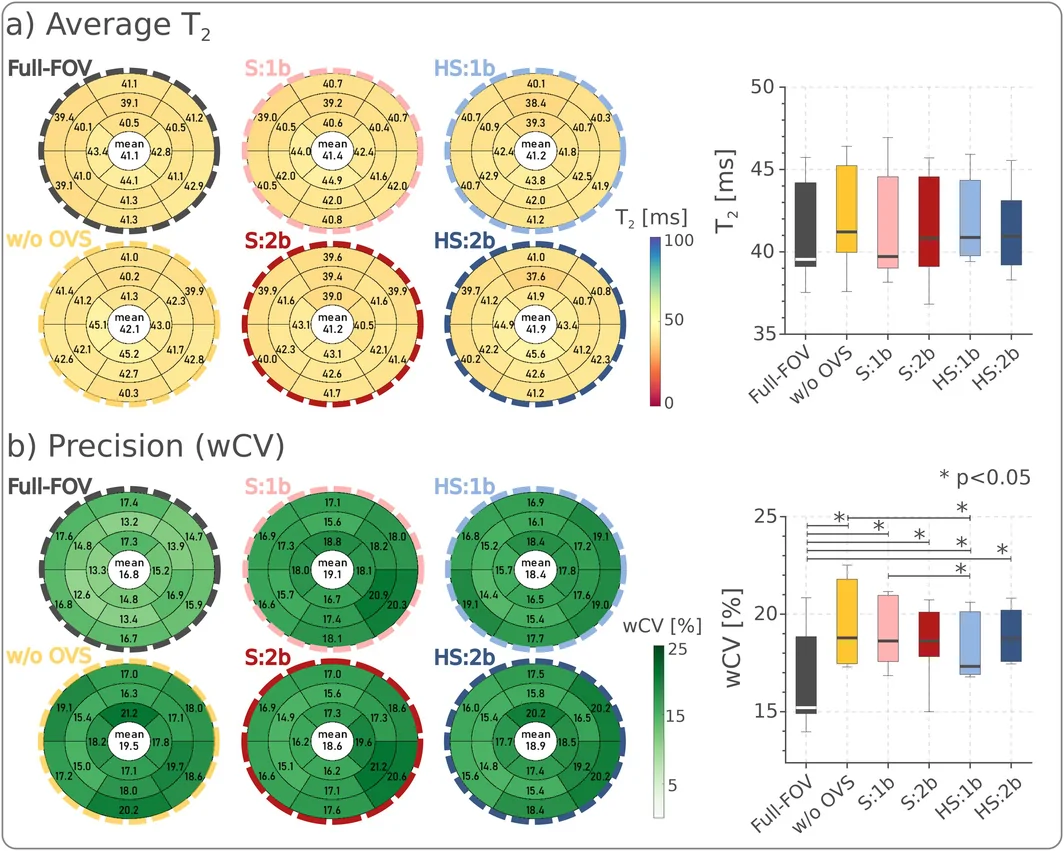
Average $T_{2}$ (a) and precision (b) of the measurements over all seven subjects across all OVS pulses and compared with full‐FOV and rFOV without OVS. Average $T_{2}$ was comparable across pulses, rFOV w/o OVS and full‐FOV, and no significant difference was observed (*p* = 0.087). Additionally, significant differences in $T_{2}$ measurement precision were observed between full‐FOV and rFOV (with and w/o OVS), between rFOV without OVS and HS:1b, as well as between S:1b and HS:1b.

The average myocardial $T_{2}$ across the seven subjects is summarized in 16 AHA segments in Figure 8a. Group‐wise assessments showed no significant differences for measured $T_{2}$ in the presence and absence of OVS (*p* = 0.087). Figure 8b shows the bullseye plot for $T_{2}$ precision averaged over seven subjects. As expected from the lower resolution, full‐FOV demonstrated the best precision (16.67% ± 2.63%) with significant differences compared with all high resolution rFOV acquisitions (*p* = 0.02). Among rFOV methods, significant differences were observed between rFOV without OVS and HS:1b (*p* = 0.015), and between HS:1b and S:1b (*p* = 0.015).

## 4 Discussion

This study investigated four different pulse classes for OVS in cardiac MRI. Suppression was optimized by tailoring suppression‐band FA to the imaging sequence and tissue $T_{1}/T_{2}$ in the suppression area, and by adapting the pulse parameters to achieve optimal suppression‐bands. Our results indicated that both single‐ and dual‐band AM‐FM pulses outperform AM pulses in terms of slice profile and suppression efficiency. AM‐FM pulses achieved the lowest residual signal in the suppression‐bands and the least amount of fold‐over artifacts in vivo.

Simulations indicated $B_{1}^{+}$ sensitivity was comparable across all pulse classes with optimized parameters, with the AM‐FM pulses being only marginally less sensitive. The $B_{1}^{+}$ sensitivity of AM‐FM pulses stems from the pulses being driven in a sub‐adiabatic regime to achieve the desired suppression‐band FA. Nonetheless, AM‐FM pulses showed higher resilience against $B_{0}$ inhomogeneity, which is consistent with their higher bandwidth. Phantom experiments revealed that AM‐FM pulses suppressed the signal more thoroughly due to the better homogeneity of their slice profiles. Dual‐band pulses for both AM and AM‐FM pulses performed slightly better in short $T_{1}$, achieving more homogeneous suppression across the two suppression‐bands.

When OVS was applied to rFOV imaging in $T_{2}$ mapping, the quantification showed good agreement with the full‐FOV reference across all pulse classes. No significant differences were observed in group‐wise comparison between the full‐FOV and rFOV approaches, despite visually apparent artifacts in the rFOV images without OVS or AM pulses. This is likely due to the relatively small and spatially confined extent of the artifact in the healthy volunteers. In patients with larger amounts of subcutaneous fat, the artifacts are expected to be of a larger extent and more disruptive to the clinical map quality. Nevertheless, the measurement precision was more sensitive to the acquisition method, with significant differences observed between the full‐FOV and all rFOV configurations (with and without OVS). Among the rFOV acquisitions, significant differences were observed between those without OVS and with HS:1b, as well as between S:1b and HS:1b. Both findings further support that AM‐FM pulses outperformed AM pulses in mitigating artifacts.

Simultaneous suppression of both suppression‐bands can be explored to create time‐efficient OVS pulses with improved homogeneity in the suppression across the two bands. Previous studies have proposed cosine modulation of AM pulses (Hamming‐windowed SLR pulses) to simultaneously suppress unwanted signals on both sides in the PE direction [3, 10]. In one study, effective OVS was achieved with dual‐band AM pulses in rFOV imaging without additional SAR compared with full‐FOV [3]. Our results suggest that dual‐band AM‐FM pulses may also be explored for OVS, offering better selectivity and bandwidth than AM pulses. For the dual‐band AM‐FM pulse, discordant FM sweeps were employed to prevent rapid phase variations in the waveform [29], which may otherwise degrade suppression‐profile accuracy if the scanner hardware imposes limitations on the achievable rate of phase change. Although the FM modulation introduces a frequency‐dependent cosine modulation, the symmetry between the two bands ensures that the resulting pulse maintains a constant phase similar to AM pulses. However, this configuration results in a time‐varying inter‐band frequency separation, which can potentially become problematic when the two bands are in close proximity. The phantom and in vivo results in this study show no significant difference in pass‐ or suppression‐band performance for concordant or discordant sweeps on the employed scan system. This suggests discordant sweeps as the more robust choice, being widely applicable also on older scan systems. In this study, parameter optimization accounting for the maximum achievable $B_{1}^{+}$ did not limit the multi‐band pulse generation. RF peak shifting has previously been proposed for AM pulses to mitigate $B_{1}^{+}$ peaks in multi‐band pulses [30]. A similar approach could be explored for AM‐FM pulses if further mitigation of $B_{1}^{+}$ peak power is required.

Deposition of RF energy at off‐resonance frequencies may elicit meaningful magnetization transfer effects, especially for AM‐FM pulses. Accordingly, the resulting pass‐band modulation (PBM) showed a signal reduction in the myocardium across all pulse types. AM‐FM pulses demonstrated a greater PBM value compared with AM pulses. Nonetheless, the MT‐induced signal loss of up to 5% across all pulses translates to minimal SNR loss, which is typically well tolerated in clinical imaging. In cases where the MT‐induced signal loss becomes relevant for the image or quantification quality, trade‐offs between increased pulse duration and overall off‐resonance energy deposition warrant further investigation.

For OVS applications, sensitivity to $B_{1}^{+}$ and $B_{0}$ inhomogeneity is the primary pulse design criterion. The simulation results indicate that this sensitivity remains comparable across different AM pulses, including linear‐phase SLR‐designed pulse, regardless of suppression‐band sharpness. This suggests that for OVS, the choice of the exact AM pulse shape is of secondary importance. Consequently, the sinc pulse was selected as a representative AM pulse for the complete in vivo comparison.

The SLR quadratic‐phase pulse [3, 5] provides an alternative means of exploiting the degrees of freedom in AM‐FM pulse design for controlled spatial excitation. The results in this study indicate that despite the different pulse principle, the suppression performance closely matched the performance of HS pulses, albeit at a higher bandwidth. However, unlike HS pulses, the suppression‐band plateau for quadratic‐phase pulses is subject to distortion in the presence of $B_{1}^{+}$ inhomogeneities [31]. This can potentially compromise the suppression efficiency and homogeneity. Additionally, the dual‐band extension, QP:2b, introduced noticeable pass‐band ripples, compromising the rFOV image quality. Nonetheless, quadratic‐phase pulses constitute an interesting alternative for realizing arbitrary suppression‐band FA with AM‐FM pulses, achieving closely matched performance to HS when applied as a single‐band pulse. The trade‐off between increased bandwidth and suppression‐band distortion in the presence of $B_{1}^{+}$ inhomogeneities warrants consideration of QP pulses if $B_{0}$ effects outweigh $B_{1}^{+}$ concerns. Furthermore, Schulte et al. [12] have shown that polynomial‐phase functions outperform quadratic approaches through steeper duration‐dependent scaling. This can be particularly beneficial for short pulse durations. Thus, a comparison between SLR polynomialphase pulses and sub‐adiabatic HS pulses warrants further investigation for OVS applications, where the pulse duration is limited.

This work focused on Cartesian imaging, as typically used in clinical MRI. In Cartesian imaging, PE is one‐dimensional. Thus, 1D‐OVS pulses can readily suppress all signals that may otherwise cause fold‐over in rFOV imaging. Multidimensional OVS pulses have been proposed to suppress the signal in multiple dimensions, to prevent aliasing in non‐Cartesian imaging. For example, 2D‐OVS was used to enable up to 5‐fold acceleration in spiral first‐pass perfusion imaging [2, 32, 33]. While our study demonstrated superior AM‐FM pulse performance over conventional AM pulses, 2D AM‐FM implementation is challenging due to the extensive pulse duration [34]. On the other hand, 1D AM‐FM could also be employed with orthogonal excitation and refocusing for 2D delineation [35, 36]. In future work, this can be integrated in preparation modules, such as $T_{2}$ preparation similar to previous studies [37].

Simultaneous multi‐slice (SMS) imaging is increasingly gaining attention in MRI for scan time acceleration based on coil sensitivities. However, the adoption of SMS in cardiac imaging [38, 39, 40, 41, 42] is hindered by the fact that regions of suboptimal coil sensitivity often overlap with bright signals from the chest and back, leading to pronounced leakage artifacts in the reconstructed images [38]. These artifacts become especially severe at higher acceleration rates and when SMS is used together with parallel imaging for in‐plane acceleration. OVS can be a powerful tool to alleviate these artifacts and facilitate higher acceleration rates in SMS. A previous study [43] has demonstrated that interleaved OVS in SMS sequences can effectively reduce leakage, enabling acceleration factors up to 10 for cine and 16 for perfusion imaging [43]. The combination of AM–FM pulses with SMS imaging may, thus, be of interest to achieve thorough leakage artifact suppression, even at high SMS rates.

This study has multiple limitations. First, the suppression‐band was optimized specifically for fat $T_{1}$, which was effective in the phantom, where only one $T_{1}$ component existed. In vivo, however, the presence of extra‐cardiac tissue with varying $T_{1}$ resulted in a larger residual signal. Combining OVS with fat‐suppression techniques such as Dixon could further reduce residual signal. Second, the current study evaluated pulse performance solely for high‐resolution myocardial $T_{2}$ mapping, while applications with other imaging sequences warrant further investigation. Finally, this study was limited by a small sample size and the inclusion of healthy volunteers only. Follow‐up investigations should expand to larger cohorts and incorporate patients with considerable subcutaneous fat to assess robustness in more clinically representative settings.

## 5 Conclusions

AM‐FM pulses outperformed AM pulses for OVS of extra‐cardiac signals. Single‐band pulses showed marginally worse homogeneity across the two suppression‐bands compared with dual‐band due to differential relaxation. The application in rFOV for myocardial $T_{2}$ mapping shows the potential to leverage OVS for increased imaging resolution. Future studies should focus on applications with SMS imaging, where OVS can be critical to mitigate inter‐slice leakage artifacts.

## Funding

This work was partially supported by Nederlandse Organisatie voor Wetenschappelijk Onderzoek, Grant/Award Number: STU.019.024; the 4TU Precision Medicine program supported by High Tech for a Sustainable Future; Nederlandse Hartstichting, Grant/Award Number: 03‐004‐2022‐0079; European Research Council, Grant/Award Number: 101078711; LUMC‐TU Delft Delft Health Initiative.

## Conflicts of Interest

Sebastian Weingärtner declares the following conflicts of interest: Founder of HiFi Imaging Inc. Hildo J. Lamb declares the following conflict of interest: Consultant for Royal Philips. Alexandru Cernicanu declares the following conflict of interest: Philips Employee. The other authors declare no conflicts of interest.

## Supporting information


**Figure S1:** Flowchart of the SLR quadratic‐phase pulse design and optimization. Parameters κ, *N*, and TBW were optimized under a peak $B_{1}^{+}$ constraint of 13.5 μT for QP:1b and 6.75 μT per band for QP:2b, with maximum similarity to the ideal slice profile. QP:2b was obtained by summing QP:1b with a frequency‐shifted copy of itself. Pulse shapes with optimized parameters (κ, *TBW*, *N*) = (−120, 90, 500) and (−139, 70, 500) for QP:1b and QP:2b are shown in the bottom panel.
**Figure S2:** (a) Four window functions used for truncation of AM sinc pulses: rectangular, Hamming, Blackman‐Harris, and Gaussian. (b) Pulse shapes obtained after applying each window function. (c) Suppression‐band profiles for all four pulses, illustrating the reduction in side‐lobes achieved through windowing. (d) Effective $M_{z}$ magnetization after sinc pulses with different window functions for various $B_{1}^{+}$ scaling. The rate of change is comparable across all four window functions, indicating similar $B_{1}^{+}$ sensitivity.
**Figure S3:** (a) Pulse shapes for the SLR design using least‐squares, maximum‐phase, and minimum‐phase filter types. (b) $B_{1}^{+}$ scaling sensitivity for all filter types, demonstrating that RF scaling not only alters the flip angle but also deteriorates suppression‐band profile homogeneity. The degree of sensitivity to $B_{1}^{+}$ scaling is comparable across all filter types.
**Figure S4:** (a) Pulse performance of QP pulses in the presence of $B_{1}^{+}$ scaling. Exacerbated suppression‐band distortion can be observed for increased $B_{1}^{+}$ scaling, particularly towards the edges of the suppression‐band. Dual‐band QP shows substantial ripple artifacts in the suppression‐band profile. (b) QP pulse performance in the presence of off‐resonance, demonstrating higher resilience of the single‐band QP pulse due to the increased bandwidth.
**Figure S5:** Comparison of suppression‐band profiles for the single‐band (QP:1b) and dualband (QP:2b) SLR quadratic‐phase designs with the remaining pulse types investigated in this study. QP pulses achieved residual signals that closely match the HS pulse performance. QP:2b demonstrated improved inter‐band homogeneity relative to QP:1b. However, passband disruption consistent with ripples observed in simulation results, remains present in the suppression‐band of the multi‐band pulse QP:2b.
**Figure S6:** (a) Suppression‐band profiles and residual signal of SLR QP pulses alongside sinc and HS pulses. QP pulses achieved residual signal comparable to HS pulses, except for residual pass‐band disruption observed with QP:2b.
**Figure S7:** (a) HS dual‐band Pulse shapes for concordant and discordant FM sweeps. (b) Suppression‐band profile and residual signal in phantom, showing closely matched performance for both sweep directions.
**Figure S8:** (a) Suppression‐band profiles and residual signals of HS dual‐band pulses with concordant and discordant FM sweeps for a representative volunteer. The residual signal magnitude is comparable between the two sweep approaches, with no appreciable disruption to the pass‐band signal observed for either FM sweep direction. (b) $T_{2}$ maps for concordant and discordant FM sweep configurations compared with the full FOV reference, shown for a representative volunteer. Both HS:2b designs yield $T_{2}$ maps closely matching the reference.

## Data Availability

All data and scripts underlying the results and figures in this manuscript can be found at: https://gitlab.tudelft.nl/mars‐lab/ovs‐t2map‐ayda.

## References

[1] J. R. Arnold and G. P. McCann , “Cardiovascular Magnetic Resonance: Applications and Practical Considerations for the General Cardiologist,” Heart 106, no. 3 (2020): 174–181. 31826937 10.1136/heartjnl-2019-314856

[2] T. B. Smith and K. S. Nayak , “Reduced Field of View MRI With Rapid, B1‐Robust Outer Volume Suppression,” Magnetic Resonance in Medicine 67, no. 5 (2012): 1316–1323. 22083545 10.1002/mrm.23116

[3] M. J. Jang , A. Gupta , A. Kovanlikaya , J. E. Scholl , and Z. Zun , “High‐Resolution Anatomical Imaging of the Fetal Brain With a Reduced Field of View Using Outer Volume Suppression,” Magnetic Resonance in Medicine 92, no. 4 (2024): 1556–1567. 38702999 10.1002/mrm.30147 PMC11262973

[4] J. P. Felmlee and R. L. Ehman , “Spatial Presaturation: A Method for Suppressing Flow Artifacts and Improving Depiction of Vascular Anatomy in MR Imaging,” Radiology 164, no. 2 (1987): 559–564. 3602402 10.1148/radiology.164.2.3602402

[5] P. Le Roux , R. J. Gilles , G. C. McKinnon , and P. G. Carlier , “Optimized Outer Volume Suppression for Single‐Shot Fast Spin‐Echo Cardiac Imaging,” Journal of Magnetic Resonance Imaging 8, no. 5 (1998): 1022–1032. 9786138 10.1002/jmri.1880080505

[6] Y. Luo , R. A. Graaf , L. DelaBarre , A. Tannús , and M. Garwood , “BISTRO: An Outer‐Volume Suppression Method That Tolerates RF Field Inhomogeneity,” Magnetic Resonance in Medicine: An Official Journal of the International Society for Magnetic Resonance in Medicine 45, no. 6 (2001): 1095–1102. 10.1002/mrm.1144 11378888

[7] B. J. Wilm , J. Svensson , A. Henning , K. P. Pruessmann , P. Boesiger , and S. S. Kollias , “Reduced Field‐of‐View MRI Using Outer Volume Suppression for Spinal Cord Diffusion Imaging,” Magnetic Resonance in Medicine: An Official Journal of the International Society for Magnetic Resonance in Medicine 57, no. 3 (2007): 625–630. 10.1002/mrm.21167 17326167

[8] D. D. Doddrell , G. J. Galloway , W. M. Brooks , et al., “The Utilization of Two Frequency‐Shifted Sinc Pulses for Performing Volume‐Selected In Vivo NMR Spectroscopy,” Magnetic Resonance in Medicine 3, no. 6 (1986): 970–975. 3821473 10.1002/mrm.1910030620

[9] S. Singh and R. Deslauriers , “Tailored Selective NMR Excitation by Projection Presaturation,” Concepts in Magnetic Resonance 7, no. 1 (1995): 1–27.

[10] A. B. Holbrook , J. M. Santos , E. Kaye , V. Rieke , and K. B. Pauly , “Real‐Time MR Thermometry for Monitoring HIFU Ablations of the Liver,” Magnetic Resonance in Medicine: An Official Journal of the International Society for Magnetic Resonance in Medicine 63, no. 2 (2010): 365–373. 10.1002/mrm.22206 PMC3212435 19950255

[11] L. Pisani , R. Bammer , and G. Glover , “Restricted Field of View Magnetic Resonance Imaging of a Dynamic Time Series,” Magnetic Resonance in Medicine 57, no. 2 (2007): 297–307. 17260360 10.1002/mrm.21115

[12] R. F. Schulte , A. Henning , J. Tsao , P. Boesiger , and K. P. Pruessmann , “Design of Broadband RF Pulses With Polynomial‐Phase Response,” Journal of Magnetic Resonance 186, no. 2 (2007): 167–175. 17331765 10.1016/j.jmr.2007.02.004

[13] I. Tkáč , Z. Starčuk , I.‐Y. Choi , and R. Gruetter , “In Vivo 1H NMR Spectroscopy of Rat Brain at 1 Ms Echo Time,” Magnetic Resonance in Medicine: An Official Journal of the International Society for Magnetic Resonance in Medicine 41, no. 4 (1999): 649–656. 10.1002/(sici)1522-2594(199904)41:4<649::aid-mrm2>3.0.co;2-g 10332839

[14] J. Luo , N. O. Addy , R. R. Ingle , et al., “Combined Outer Volume Suppression and T2 Preparation Sequence for Coronary Angiography,” Magnetic Resonance in Medicine 74, no. 6 (2015): 1632–1639. 25521477 10.1002/mrm.25575 PMC4470881

[15] D. C. Shungu and J. D. Glickson , “Sensitivity and Localization Enhancement in Multinuclear In Vivo NMR Spectroscopy by Outer Volume Presaturation,” Magnetic Resonance in Medicine 30, no. 6 (1993): 661–671. 8139447 10.1002/mrm.1910300603

[16] J. Z. Bojorquez , S. Bricq , C. Acquitter , F. Brunotte , P. M. Walker , and A. Lalande , “What Are Normal Relaxation Times of Tissues at 3 T?,” Magnetic Resonance Imaging 35 (2017): 69–80. 27594531 10.1016/j.mri.2016.08.021

[17] J. M. Warnking and G. B. Pike , “Bandwidth‐Modulated Adiabatic RF Pulses for Uniform Selective Saturation and Inversion,” Magnetic Resonance in Medicine: An Official Journal of the International Society for Magnetic Resonance in Medicine 52, no. 5 (2004): 1190–1199. 10.1002/mrm.20262 15508169

[18] A. T. O'Brien , K. E. Gil , J. Varghese , O. P. Simonetti , and K. M. Zareba , “T2 Mapping in Myocardial Disease: A Comprehensive Review,” Journal of Cardiovascular Magnetic Resonance 24, no. 1 (2022): 33. 35659266 10.1186/s12968-022-00866-0 PMC9167641

[19] R. J. Ogg , R. B. Kingsley , and J. S. Taylor , “WET, a T1‐and B1‐Insensitive Water‐Suppression Method for In Vivo Localized 1H NMR Spectroscopy,” Journal of Magnetic Resonance. Series B 104, no. 1 (1994): 1–10. 8025810 10.1006/jmrb.1994.1048

[20] M. Akçakaya , T. A. Basha , S. Weingärtner , S. Roujol , S. Berg , and R. Nezafat , “Improved Quantitative Myocardial T2 Mapping: Impact of the Fitting Model,” Magnetic Resonance in Medicine 74, no. 1 (2015): 93–105. 25103908 10.1002/mrm.25377 PMC4320682

[21] R. M. Henkelman , “Magnetization Transfer in MRI: A Review,” NMR in Biomedicine 14 (2001): 57–64. 11320533 10.1002/nbm.683

[22] S. D. Wolff and R. S. Balaban , “Magnetization Transfer Contrast (MTC) and Tissue Water Proton Relaxation In Vivo,” Magnetic Resonance in Medicine 10, no. 1 (1989): 135–144. 2547135 10.1002/mrm.1910100113

[23] N. I. Niebuhr , W. Johnen , G. Echner , et al., “The ADAM‐Pelvis Phantom—An Anthropomorphic, Deformable and Multimodal Phantom for MRgRT,” Physics in Medicine & Biology 64, no. 4 (2019): 04NT05. 10.1088/1361-6560/aafd5f 30630152

[24] Q. Tao , P. Tol , F. F. Berendsen , E. H. M. Paiman , H. J. Lamb , and R. J. Geest , “Robust Motion Correction for Myocardial T1 and Extracellular Volume Mapping by Principle Component Analysis‐Based Groupwise Image Registration,” Journal of Magnetic Resonance Imaging 47, no. 5 (2018): 1397–1405. 28960659 10.1002/jmri.25863

[25] Y. Zhang , Y. Zhao , L. Huang , L. Xia , and Q. Tao , “Deep‐Learning‐Based Groupwise Registration for Motion Correction of Cardiac T1 Mapping,” in International Conference on Medical Image Computing and Computer‐Assisted Intervention (IEEE, 2024).

[26] Y. Zhao , P. Kellman , H. Xue , et al., “Reverse Imaging for Wide‐Spectrum Generalization of Cardiac MRI Segmentation,” in International Conference on Medical Image Computing and Computer‐Assisted Intervention (Springer, 2025), 555–565.

[27] Y. Zhao , C. Yang , A. Schweidtmann , and Q. Tao , “Efficient Bayesian Uncertainty Estimation for nnU‐Net,” in International Conference on Medical Image Computing and Computer‐Assisted Intervention (IEEE, 2022), 535–544.

[28] Y. Zhao , J. Tourais , I. Pierce , et al., “Bayesian Uncertainty Estimation by Hamiltonian Monte Carlo: Applications to Cardiac MRI Segmentation,” Machine Learning for Biomedical Imaging 2 (2024): 856–887.

[29] E. Kupce and R. Freeman , “Close Encounters Between Soft Pulses,” Journal of Magnetic Resonance, Series A 112, no. 2 (1995): 261–264.

[30] E. J. Auerbach , J. Xu , E. Yacoub , S. Moeller , and K. Uğurbil , “Multiband Accelerated Spin‐Echo Echo Planar Imaging With Reduced Peak RF Power Using Time‐Shifted RF Pulses,” Magnetic Resonance in Medicine 69, no. 5 (2013): 1261–1267. 23468087 10.1002/mrm.24719 PMC3769699

[31] R. F. Schulte , J. Tsao , P. Boesiger , and K. P. Pruessmann , “Equi‐Ripple Design of Quadratic‐Phase RF Pulses,” Journal of Magnetic Resonance 166, no. 1 (2004): 111–122. 14675826 10.1016/j.jmr.2003.10.009

[32] J. Wang , Y. Yang , D. S. Weller , et al., “High Spatial Resolution Spiral First‐Pass Myocardial Perfusion Imaging With Whole‐Heart Coverage at 3 T,” Magnetic Resonance in Medicine 86, no. 2 (2021): 648–662. 33709415 10.1002/mrm.28701

[33] Y. Yang , L. Zhao , X. Chen , et al., “Reduced Field of View Single‐Shot Spiral Perfusion Imaging,” Magnetic Resonance in Medicine 79, no. 1 (2018): 208–216. 28321908 10.1002/mrm.26664 PMC5607067

[34] S. Conolly , J. Pauly , D. Nishimura , and A. Macovsiu , “Two‐Dimensional Selective Adiabatic Pulses,” Magnetic Resonance in Medicine 24, no. 2 (1992): 302–313. 1569869 10.1002/mrm.1910240211

[35] D. A. Feinberg , J. C. Hoenninger , L. E. Crooks , L. Kaufman , J. C. Watts , and M. Arakawa , “Inner Volume MR Imaging: Technical Concepts and Their Application,” Radiology 156, no. 3 (1985): 743–747. 4023236 10.1148/radiology.156.3.4023236

[36] F. Gibiino , S. Lechner‐Greite , T. Schirmer , et al., “Effects of Inner Volume Field‐of‐View Reduction on Myocardial T2 Mapping,” Journal of Magnetic Resonance Imaging 42, no. 1 (2015): 175–179. 25256847 10.1002/jmri.24763

[37] S. Soleimanifard , M. Schär , A. G. Hays , J. L. Prince , R. G. Weiss , and M. Stuber , “Spatially Selective Implementation of the Adiabatic T2Prep Sequence for Magnetic Resonance Angiography of the Coronary Arteries,” Magnetic Resonance in Medicine 70, no. 1 (2013): 97–105. 22915337 10.1002/mrm.24437 PMC3530637

[38] S. Weingärtner , S. Moeller , S. Schmitter , et al., “Simultaneous Multislice Imaging for Native Myocardial T1 Mapping: Improved Spatial Coverage in a Single Breath‐Hold,” Magnetic Resonance in Medicine 78, no. 2 (2017): 462–471. 28580583 10.1002/mrm.26770 PMC5509494

[39] Y. Yang , C. H. Meyer , F. H. Epstein , C. M. Kramer , and M. Salerno , “Whole‐Heart Spiral Simultaneous Multi‐Slice First‐Pass Myocardial Perfusion Imaging,” Magnetic Resonance in Medicine 81, no. 2 (2019): 852–862. 30311689 10.1002/mrm.27412 PMC6289615

[40] Z. Bentatou , T. Troalen , M. Bernard , et al., “Simultaneous Multi‐Slice T1 Mapping Using MOLLI With Blipped CAIPIRINHA bSSFP,” Magnetic Resonance Imaging 95 (2023): 90–102. 32304799 10.1016/j.mri.2020.03.006

[41] Y. Tian , S. X. Cui , Y. Lim , N. G. Lee , Z. Zhao , and K. S. Nayak , “Contrast‐Optimal Simultaneous Multi‐Slice bSSFP Cine Cardiac Imaging at 0.55 T,” Magnetic Resonance in Medicine 89, no. 2 (2023): 746–755. 36198043 10.1002/mrm.29472 PMC9712243

[42] S. McElroy , G. Ferrazzi , M. S. Nazir , et al., “Simultaneous Multislice Steady‐State Free Precession Myocardial Perfusion With Full Left Ventricular Coverage and High Resolution at 1.5 T,” Magnetic Resonance in Medicine 88, no. 2 (2022): 663–675. 35344593 10.1002/mrm.29229 PMC9310832

[43] Ö. B. Demirel , S. Weingärtner , S. Moeller , and M. Akçakaya , “Improved Simultaneous Multi‐Slice Imaging for Perfusion Cardiac MRI Using Outer Volume Suppression and Regularized Reconstruction,” in 2020 IEEE 17th International Symposium on Biomedical Imaging (ISBI) (IEEE, 2020), 1954–1957.

