# Supplementary material for: Comparison of Amplitude‐ and Amplitude‐Frequency‐Modulated Pulses for Outer Volume Suppression in Cardiac MRI: Application to Myocardial T2 Mapping
Source: Magn Reson Med. 2026 Aug 6;96(5):2240–52. doi: 10.1002/mrm.70515 (PMC13527258; doi:10.1002/mrm.70515)
Supplement: Supplementary file 1 [file MRM-96-2240-s001.pdf]

SUPPLEMENTARY MATERIAL FOR:

Comparison of Amplitude- and Amplitude-Frequency modulated Pulses for Outer Volume Suppression in Cardiac MRI: Application to myocardial T_2 Mapping

Ayda Arami[1,2], Maša Božić-Iven[1], Christal van de Steeg-Henzen[3], Yidong Zhao[1], Yi Zhang[1], Qian Tao[1], Alexandru Cernicanu[4], Hildo J. Lamb[2], and Sebastian Weingärtner[1,3]

[1]Department of Imaging Physics, Delft University of Technology, Delft, The Netherlands
[2]Department of Radiology, Leiden University Medical Center, Leiden, The Netherlands
[3]Holland PTC, Delft, The Netherlands
[4]Philips Benelux, Eindhoven, The Netherlands

INDEX OF SUPPLEMENTARY MATERIALS

Methods 2
AM Pulse Window Functions . . . 2
AM Linear Phase SLR Pulses . . . 2
AM-FM Quadratic-Phase SLR Pulses . . . 2
Dual-band HS Pulses With Concordant and Discordant Frequency Modulation . . . 2

Results 2
AM Pulse Window Functions . . . 3
AM Linear Phase SLR Pulses . . . 4
AM-FM Quadratic-Phase SLR Pulses . . . 5
Dual-band HS Pulses With Concordant and Discordant Frequency Modulation . . . 5

INDEX OF SUPPLEMENTARY FIGURES AND TABLES

Figure S1 SLR Optimization flowchart . . . 3

Figure S2 AM pulse sinc truncation using different window functions. . . . 4

Figure S3 SLR designed linear phase AM pulses . . . 6

Figure S4 AM-FM Quadratic-Phase SLR Pulses - Simulation . . . 7

Figure S5 AM-FM Quadratic-Phase SLR Pulses - Phantom experiments . . . 8

Figure S6 AM-FM Quadratic-Phase SLR Pulses - In vivo experiments . . . 8

Figure S7 Concordant vs Discordant FM sweep in Dual-band HS . . . 9

Figure S8 Concordant vs Discordant FM sweep in Dual-band HS- In vivo . . . 9

Methods

AM Pulse Sinc Window Functions

AM pulse performance has been evaluated in simulations for sinc pulses subject to different window functions to study the effect of truncation. Sinc pulse was generated with 10 ms duration, 512 samples, and using a Gradient strength of 240 μT/m (the optimized Gradient for sinc with rectangular truncation). The following window functions were applied: 1) Rectangular (no windowing), Hamming, Blackman-Harris, and Gaussian ($\sigma = 3$) (FigureS2a). The slice profile was calculated for each windowed pulse, and the effective flip angle was assessed for B_1^+-scaling between 0.8 and 1.2.

AM Linear Phase SLR Pulses

Additional AM pulses were generated using the Shinnar-Le-Roux (SLR) algorithm for linear phase pulses using MATLAB R2020b (Larson 2025). Three SLR-designed pulses with least-squares (LS), minimum-phase (Min), and maximum-phase (Max) filter types were evaluated under identical design constraints: a fixed duration of 10 ms, a flip angle of 120°, and a peak B_1^+ limit of 13.5 μT. Pulses were compared in terms of slice profile, bandwidth, and sensitivity B_1^+ scaling (between 0.8 to 1.2).

AM-FM Quadratic Phase SLR Pulses

To study additional AM-FM pulses, the SLR algorithm was used to generate quadratic-phase pulses. An optimization framework, analogous to the one used for the other pulse types, was employed to identify parameters that yield a homogeneous slice profile while satisfying the hardware constraint of a maximum B_1^+ amplitude of 13.5 μT. For quadratic-phase SLR pulses, the number of samples (N), the time-bandwidth product (TBW), and the scaling parameter κ, which governs the quadratic-phase modulation, were subject to optimization. The optimization procedure is summarized in the flowchart shown in Supporting Information Figure S1. Pulse performance under B_1^+/B_0 inhomogeneity was studied using Bloch simulations. Single- (QP:1b) and dual-band (QP:2b) quadratic-phase pulses were generated and compared to the other pulse types in terms of residual signal in phantom experiments and in vivo in 4 healthy volunteers (4 Males; 26±0.81 y/o), using the same sequences and metrics as described in the main manuscript.

Dual-band HS Pulses With Concordant and Discordant Frequency Modulation

Dual-band HS pulses were generated from single-band pulses in two different ways (Supporting Information Figure S7a): 1) Simple addition of the frequency-shifted single-band HS pulses, leading to concordantly changing FM of the constituent pulses. 2) Time reversal of one of the single-band HS pulses before combination, to create a constant phase pulse, resulting from the discordant FM. Concordant and discordant dual-band HS pulses were compared in terms of pass-band and suppression-band signals in the phantom. Suppression-band profile and T_2 map quantification were evaluated in four healthy subjects (4 Males; 26±0.81 y/o). The same imaging methodology and evaluation as described in the main manuscript were used in phantom and in vivo experiments.

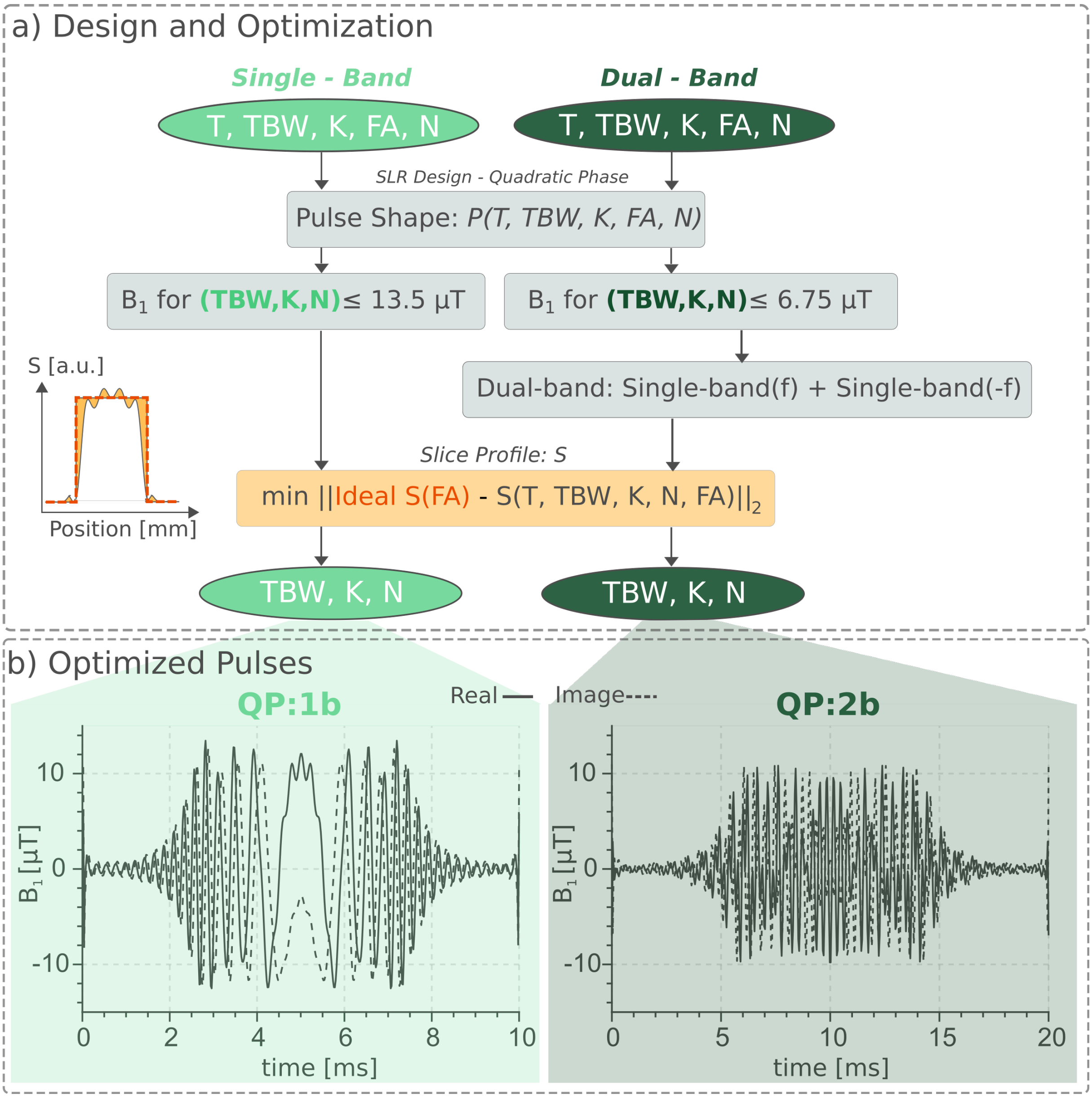

Figure S1: Flowchart of the SLR quadratic-phase pulse design and optimization. Parameters κ, N, and TBW were optimized under a peak B_1^+ constraint of 13.5 μT for QP:1b and 6.75 μT per band for QP:2b, with maximum similarity to the ideal slice profile. QP:2b was obtained by summing QP:1b with a frequency-shifted copy of itself. Pulse shapes with optimized parameters $(\kappa, TBW, N) = (-120, 90, 500)$ and $(-139, 70, 500)$ for QP:1b and QP:2b are shown in the bottom panel.

Results

AM Pulse Sinc Window Functions

Figure S2c confirms the expected reduction in side-lobe amplitude with progressive windowing. At the same time, the effective suppression-bandwidth remains essentially largely unchanged. Figure S2-d shows that the flip-angle response to B_1^+ variation is similarly window-independent, with sensitivity slopes of: Rectangular(R^2=0.99, slope=-0.15, intercept=1.45), and similar performance for Hamming, Blackman-Harris, and Gaussian (R^2=0.99, slope=-0.15, intercept=1.44). The off-resonance band shift is comparable across windows, as expected from the fixed bandwidth. Taken together, these results suggest

that, although window functions can shape the slice profile, the OVS performance of the AM pulse under B_0 and B_1^+ inhomogeneity is largely insensitive to this choice.

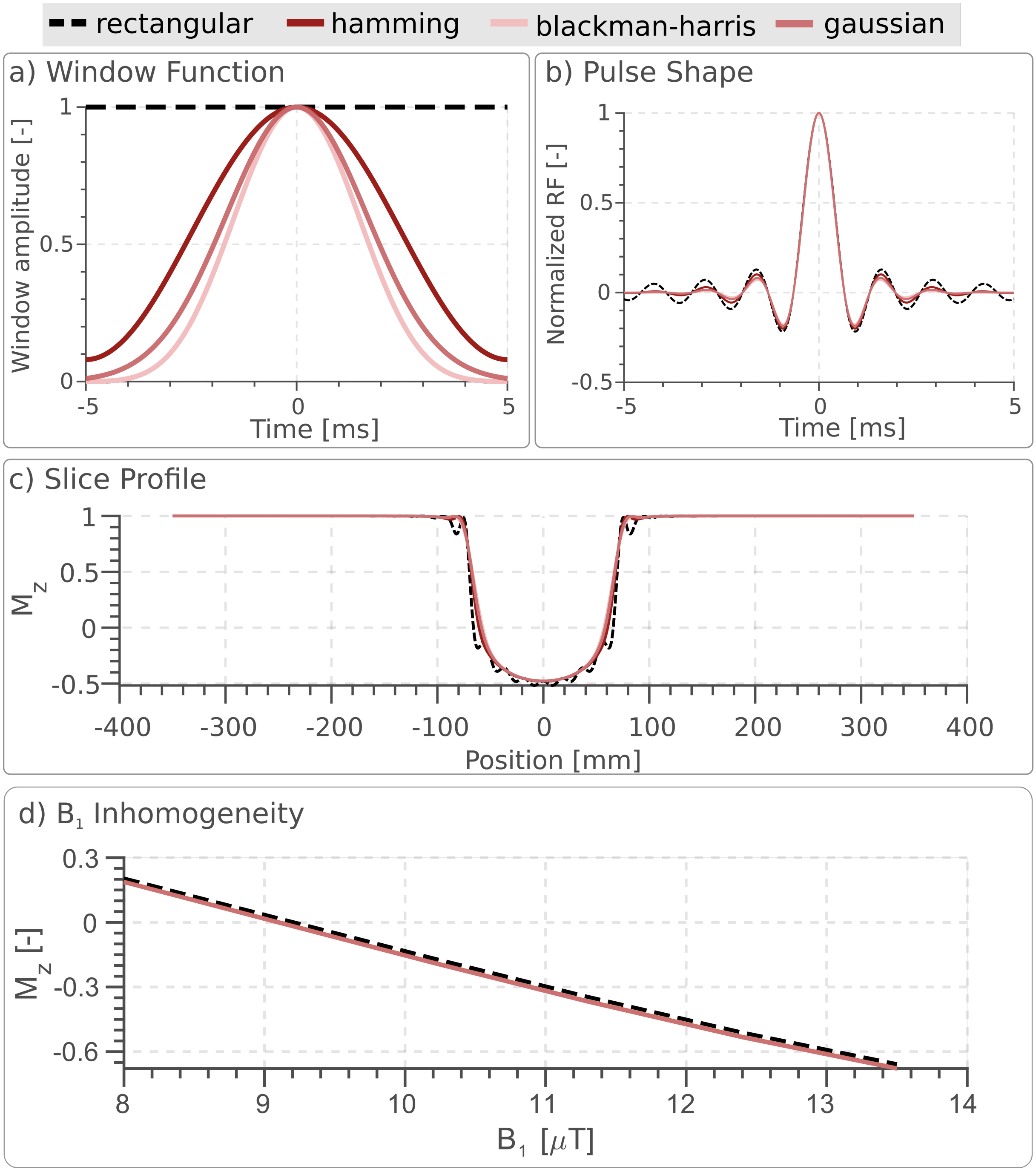

Figure S2: **a)** Four window functions used for truncation of AM sinc pulses: rectangular, Hamming, Blackman-Harris, and Gaussian. **b)** Pulse shapes obtained after applying each window function. **c)** Suppression-band profiles for all four pulses, illustrating the reduction in side-lobes achieved through windowing. **d)** Effective M_z magnetization after sinc pulses with different window functions for various B_1^+ scaling. The rate of change is comparable across all four window functions, indicating similar B_1^+ sensitivity.

AM Linear Phase SLR Pulses

The maximum achievable TBW values were 12, 14, and 14, with corresponding peak B_1^+ amplitudes of 11.78, 12.76, and 12.76 μT for LS, Max-phase, and Min-phase filters, respectively [Figure S3a]. B_1^+ scaling sensitivity was comparable across all filter types (dMz per μT: LS: -0.15, Max-phase: -0.14, Min-phase: -0.14) (Supporting Information Figure S3b), consistent with the behavior observed in sinc pulses with different window functions. The comparable TBW values further imply similar B_0 sensitivity across all three designs, in line with that of sinc pulses.

AM-FM Quadratic-Phase SLR Pulses

The optimized parameters (κ, TBW, N) for QP:1b and QP:2b were (-120, 90, 500) and (-139, 70, 500), respectively. This implies QP:1b achieves a 40.9% wider bandwidth than HS:1b. Supporting Information Figure S4-a shows that B_1^+ scaling not only alters the flip angle but also distorts the slice profile within the suppression band. The band shift under B_0 inhomogeneity is smaller for QP:1b than for QP:2b, owing to the higher bandwidth of QP:1b (Supporting Information Figure S4). Additionally, QP:2b exhibited greater pass-band distortion than QP:1b. In phantom experiments, residual signals between HS and QP pulses were closely matched (QP:1b: $12.64 \pm 7.93\%$; QP:2b: $7.27 \pm 610\%$; HS:1b: $14.13 \pm 7.94\%$; HS:2b: $9.55 \pm 5.5\%$) (Supporting Information Fig. S S5). Only QP:2b exhibited noticeable ripple artifacts resulting in signal disruption within the pass-band, as also observed in simulations. (see Supporting Information Figures S5 and S6). In vivo measurements confirm these trends, with QP:1b demonstrating residual signal in close agreement with HS:1b ($16.43 \pm 6.47\%$ vs. $17.31 \pm 6.22\%$), while QP:2b shows signal disruption in the pass-band that is consistent with the residual ripples observed in the phantom (Supporting Information Figures S5 and S6).

Dual-band HS Pulses With Concordant and Discordant Frequency Modulation

Phantom results (figure S7) showed comparable performance in both pass- and suppression-bands of the concordant and discordant HS dual-band pulse (Residual signal: concordant 10.63%; discordant 9.06%). The residual signal averaged across four volunteers was $20.14 \pm 9.94\%$ for the concordant pulse and $17.38 \pm 5.88\%$ for the discordant pulse, confirming comparable suppression performance. Mean T_2 values across four volunteers were 47.62 ± 4.78 and 47.09 ± 4.61 for the concordant and discordant pulses, respectively. Taken together, the phantom and in vivo results indicate that the effect of sweep direction on pulse performance is negligible in the context of this study.

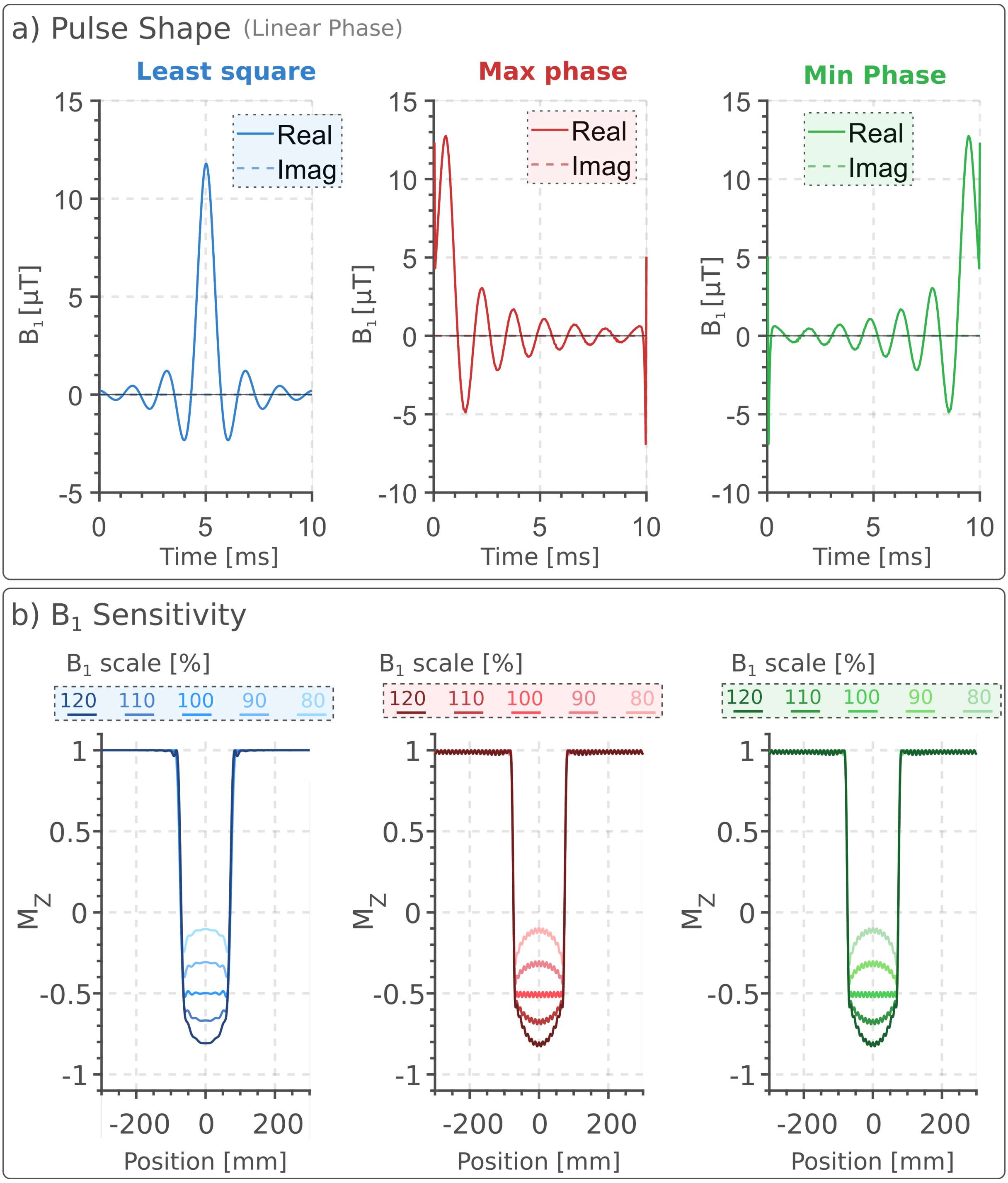

Figure S3: **a)**Pulse shapes for the SLR design using least-squares, maximum-phase, and minimum-phase filter types. **b)** B_1^+ scaling sensitivity for all filter types, demonstrating that RF scaling not only alters the flip angle but also deteriorates suppression-band profile homogeneity. The degree of sensitivity to B_1^+ scaling is comparable across all filter types.

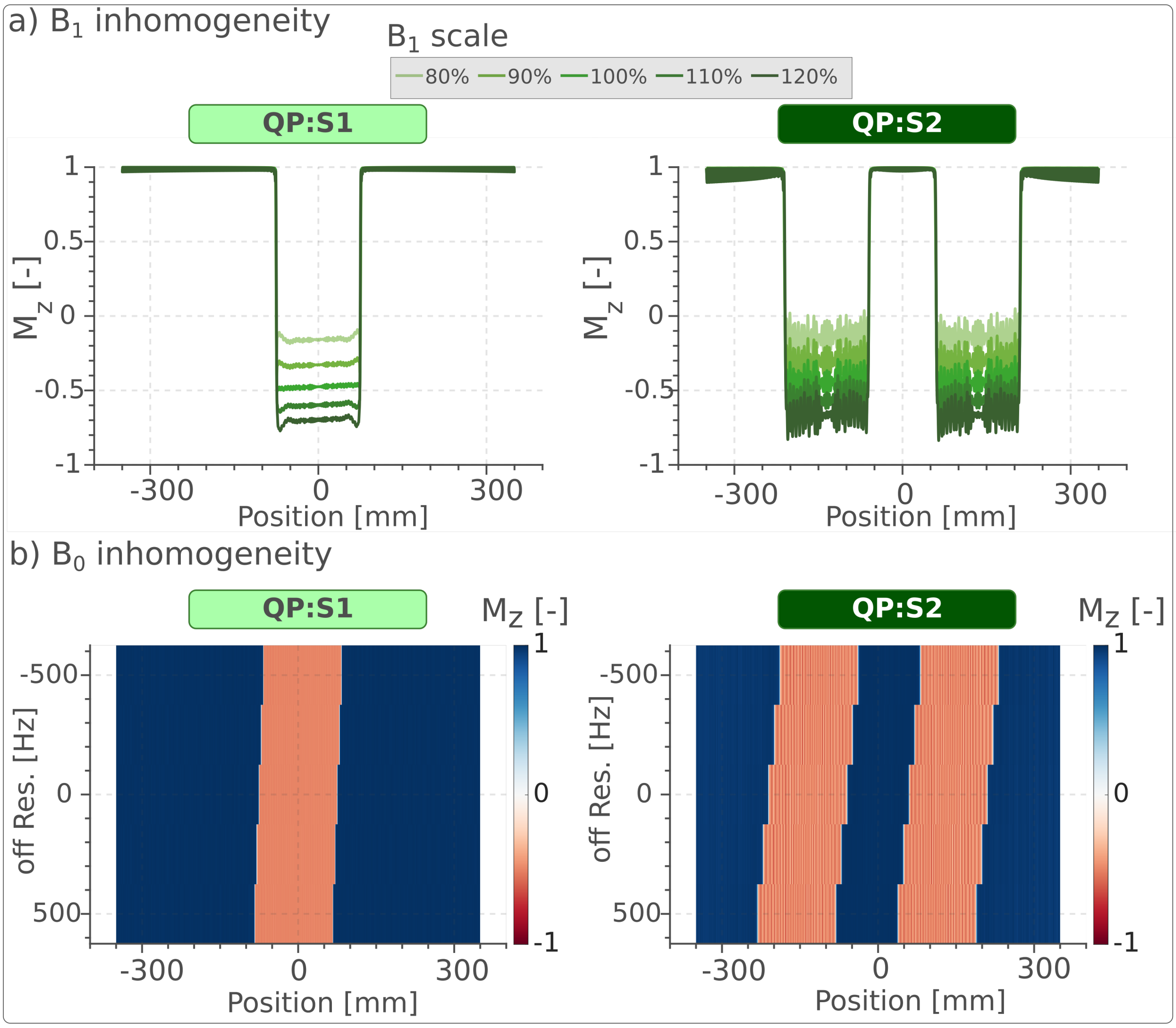

Figure S4: a) Pulse performance of QP pulses in the presence of B_1^+ scaling. Exacerbated suppression-band distortion can be observed for increased B_1^+ scaling, particularly towards the edges of the suppression-band. Dual-band QP shows substantial ripple artifacts in the suppression-band profile. **b)** QP pulse performance in the presence of off-resonance, demonstrating higher resilience of the single-band QP pulse due to the increased bandwidth.

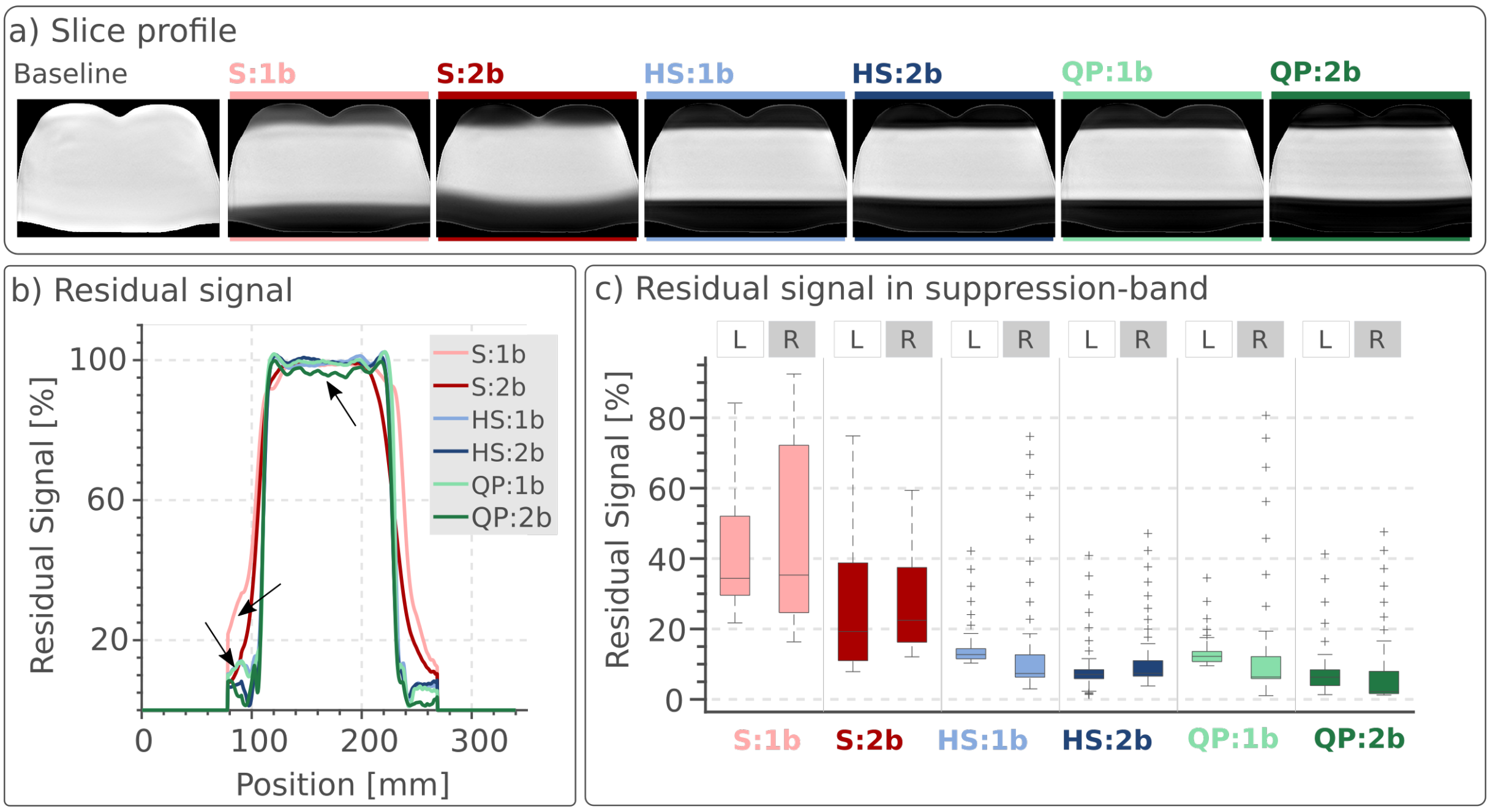

Figure S5: Comparison of suppression-band profiles for the single-band (QP:1b) and dual-band (QP:2b) SLR quadratic-phase designs with the remaining pulse types investigated in this study. QP pulses achieved residual signals that closely match the HS pulse performance. QP:2b demonstrated improved inter-band homogeneity relative to QP:1b. However, pass-band disruption consistent with ripples observed in simulation results, remains present in the suppression-band of the multi-band pulse QP:2b.

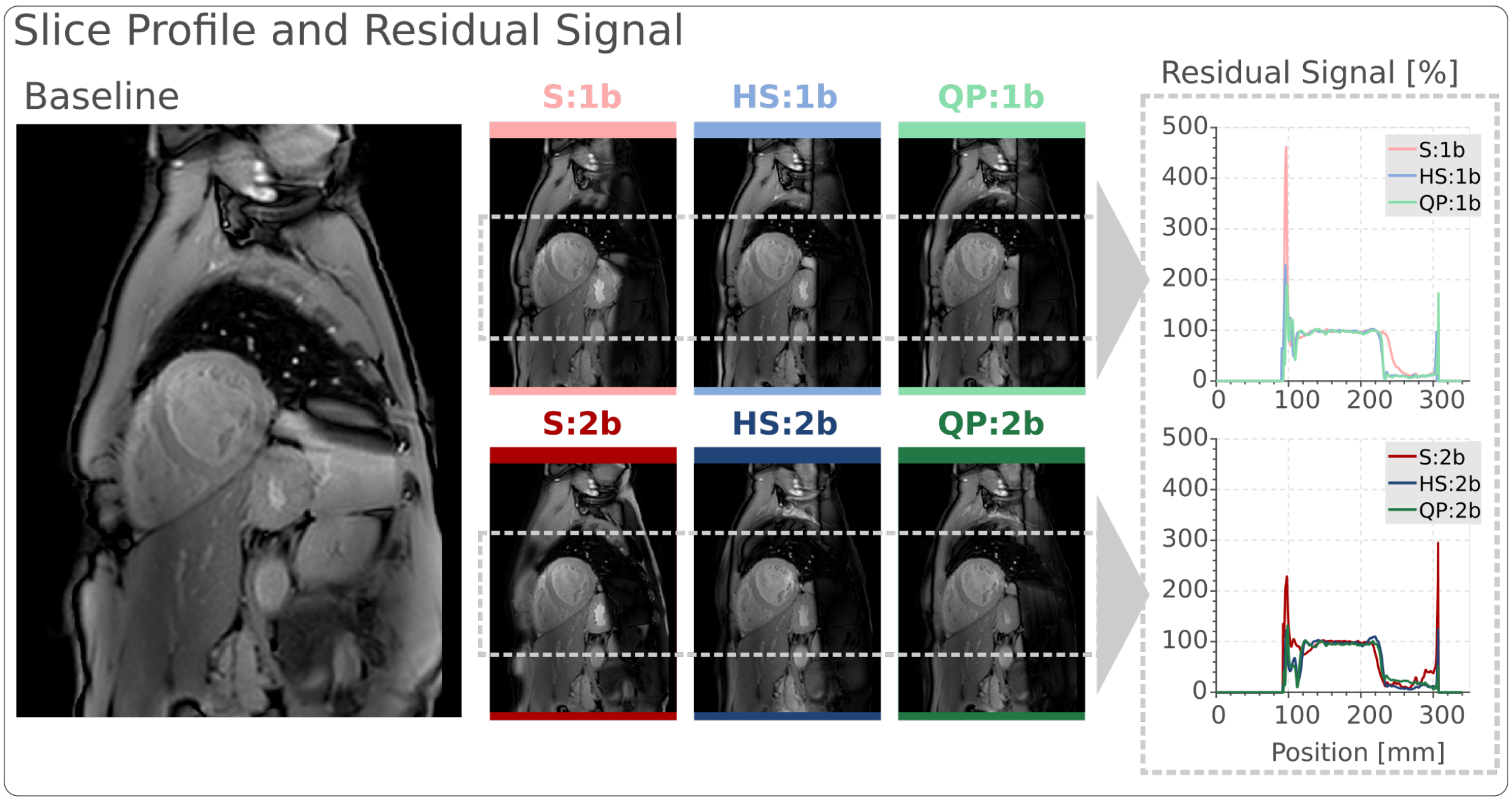

Figure S6: **a)** Suppression-band profiles and residual signal of SLR QP pulses alongside sinc and HS pulses. QP pulses achieved residual signal comparable to HS pulses, except for residual pass-band disruption observed with QP:2b.

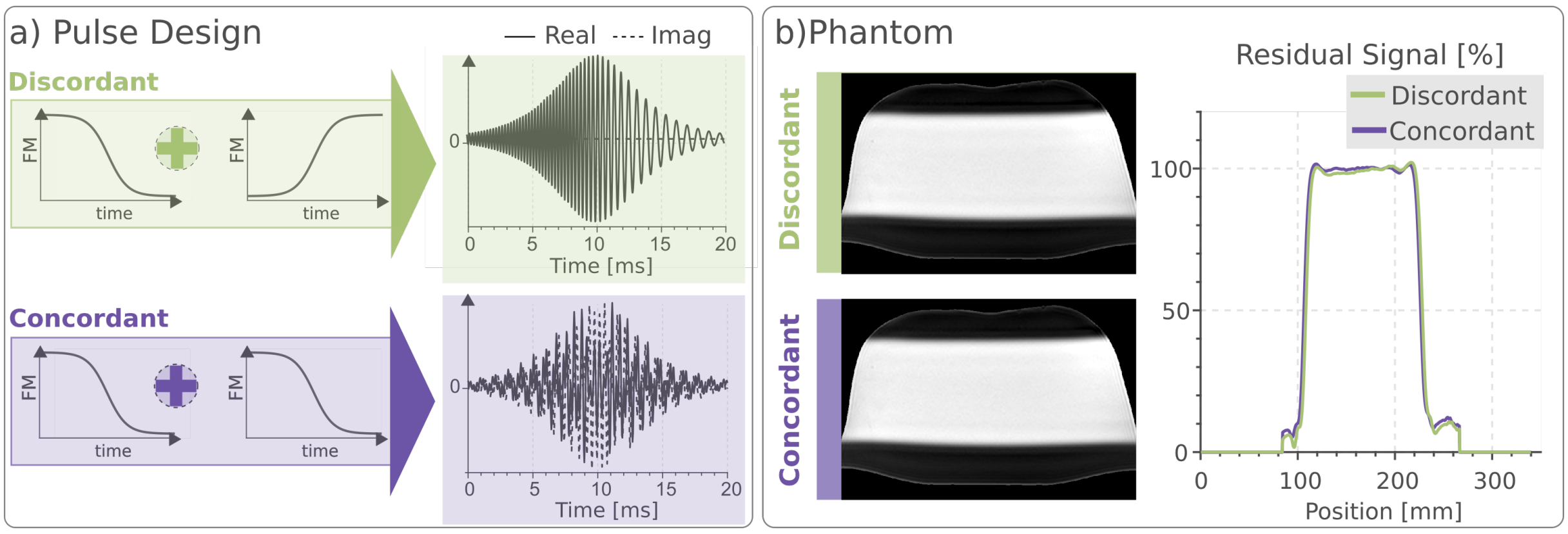

Figure S7: a) a) HS dual-band Pulse shapes for concordant and discordant FM sweeps. **b)**Suppression-band profile and residual signal in phantom, showing closely matched performance for both sweep directions.

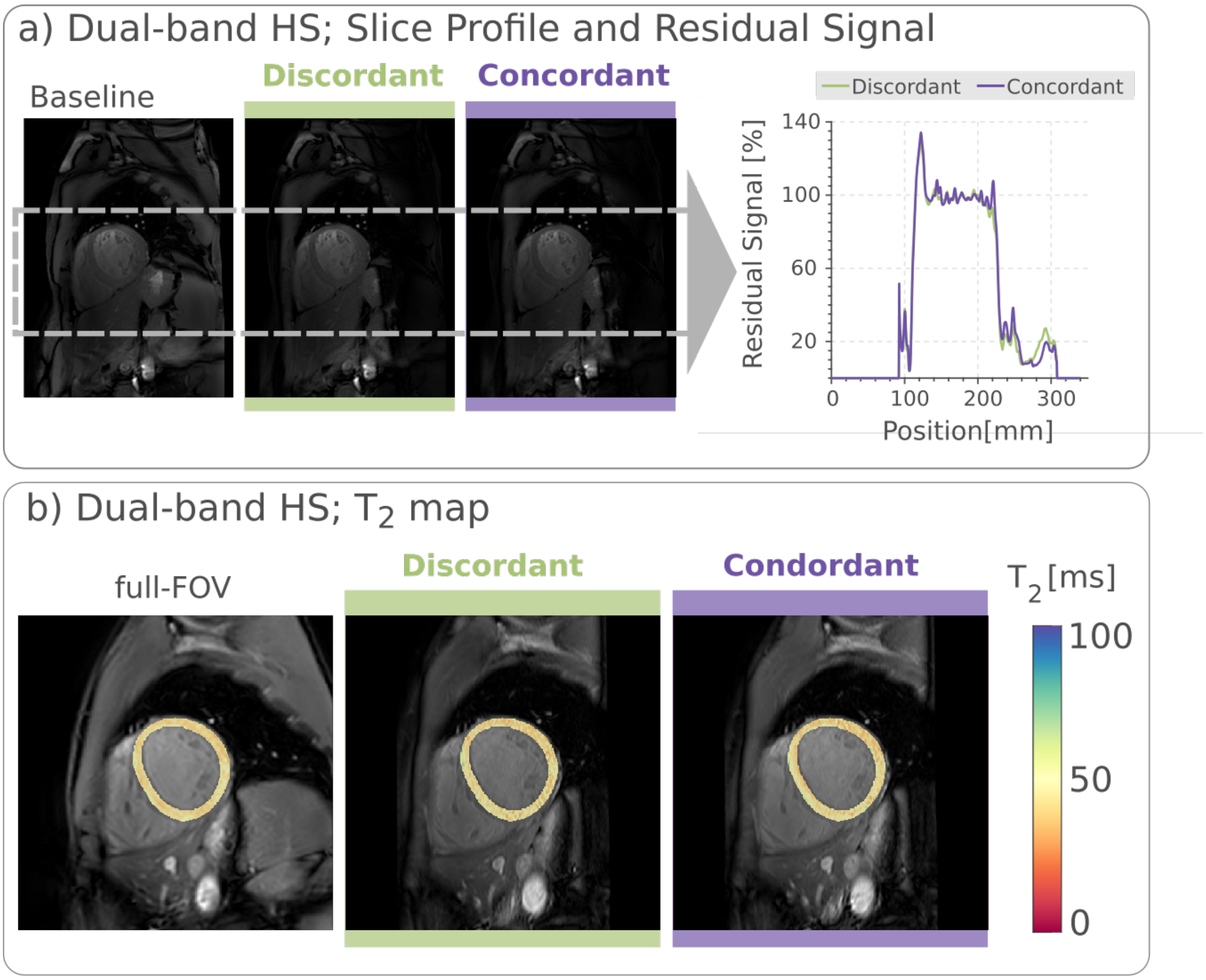

Figure S8: a) a) Suppression-band profiles and residual signals of HS dual-band pulses with concordant and discordant FM sweeps for a representative volunteer. The residual signal magnitude is comparable between the two sweep approaches, with no appreciable disruption to the pass-band signal observed for either FM sweep direction. **b)** T_2 maps for concordant and discordant FM sweep configurations compared with the full FOV reference, shown for a representative volunteer. Both HS:2b designs yield T_2 maps closely matching the reference.

References

Larson, Peder E. Z. (2025). Principles of MRI. Retrieved from https://larsonlab.github.io/MRI-education-resources. DOI: 10.5281/zenodo.5547020. URL: https://larsonlab.github.io/MRI-education-resources.
